# Paving the way for more accessible cancer care in low-income countries with optimization

**DOI:** 10.1007/s10729-026-09754-w

**Published:** 2026-09-16

**Authors:** Abel Sapirstein, Lauren N. Steimle, Mathieu Dahan

**Affiliations:** https://ror.org/01zkghx44 grid.213917.f 0000 0001 2097 4943 H. Milton Stewart School of Industrial and Systems Engineering, Georgia Institute of Technology, Atlanta, GA USA

**Keywords:** Healthcare access, Facility location, Network design, Cancer care, Geospatial analysis, Low-income countries

## Abstract

Cancers are a growing cause of morbidity and mortality in low-income countries. Geographic access plays a key role in both timely diagnosis and successful treatment. In areas lacking well-developed road networks, seasonal weather events can lengthen already long travel times to access care. Expanding facilities to offer cancer care is expensive and requires staffing by skilled medical professionals, which are often in short supply. In this article, we propose a mathematical model to improve geographic access to cancer care by jointly considering expansions to care facilities and improvements to the road network. We model this as a multi-period stochastic facility location network design problem. For each period, a decision maker must simultaneously choose a set of facilities at which to add tertiary cancer services and a set of roads to improve while facing demand and travel time uncertainty. Once demand for cancer care and weather events are realized, patients observe road conditions and use the transportation network to travel towards the closest facility with available cancer services. We create a new path-based formulation of this problem and develop a new branch-price-and-cut algorithm with acceleration techniques that take advantage of this formulation’s structure. We demonstrate our approach using Rwanda as a case study and show that the reductions in travel time to cancer care directly attributable to the road network improvements can be as high as one hour.

## Highlights

- Tertiary cancer care is expensive and often not widely available.
- Accessing this care is particularly challenging in regions with poor road conditions, especially during rainy seasons.
- We develop a multi-period stochastic optimization model that plans both the expansion of cancer care and improvements to the road network to improve access.
- We design a tailored branch-price-and-cut algorithm with custom acceleration strategies to solve large-scale real-world instances of the model.
- In a case study of Rwanda, we find that jointly improving roads and expanding care provides the biggest benefits for patients currently farthest from care.

## Introduction

### Motivation

Cancer is a leading cause of morbidity and mortality worldwide, but the impacts of cancer are not felt equally. It is estimated that 9.6 million people die from cancer each year, and that 70% of these deaths occur in low- and middle-income countries [1]. The majority of new cancer cases occur in these countries and have higher mortality than in high-income countries [2]. For instance, 6% of cancer cases globally are estimated to occur in sub-Saharan Africa, but this region accounts for 15% of cancer-attributable deaths [2]. Compared to high-income countries, there is a higher proportion of cancer patients in low-income countries (LICs) who have already progressed to an advanced stage of the disease by the time they are diagnosed [3], and consequently there is a higher case fatality rate [4]. Accordingly, early diagnosis and access to treatment play an important role in the cancer control strategy for many LICs [5].

Geographic accessibility plays a key role in early diagnosis and treatment in LICs. It is well-documented that patients with worse geographic access receive care at later stages of disease [2, 6]. However, the uneven distribution of cancer care facilities and the quality of the road network can result in extremely long travel times to access care, especially for those living in rural areas. For instance, more than 25% of Rwandans and Nigerians live more than four hours from cancer care [6–8].

Moreover, people living in rural regions are more likely to experience disruptions in access due to extreme weather events [9]. Often, roads in rural areas tend to be gravel or dirt, which makes them particularly susceptible to being washed out by heavy rainfalls [10]. Meanwhile, the available data suggest that rural regions in sub-Saharan Africa have higher rates of cancer-attributable mortality than their urban counterparts [6, 11]. Altogether, rural populations in LICs are especially impacted by a lack of geographic access to cancer care.

Given the importance of geographic access on early diagnosis and access to treatment, identifying solutions to improve access to cancer care is a crucial step in improving health outcomes and mitigating health disparities on national and global scales. Indeed, many ministries of health and non-profit organizations are looking to make investments to expand access to care [7, 12–14]. However, it remains unclear how service and infrastructure improvements can best improve access to care, particularly for rural populations when roads are severely impacted by weather events.

This leads us to the following research question: *How should health system planners allocate resources to jointly improve cancer care services and road infrastructure for maximizing access to care under demand and travel time uncertainty?*

We hypothesized that enhancing the roads on which patients travel in concert with expanding services could significantly improve access to cancer care, especially for currently under-served patients.

### Contributions

In this article, we describe a new approach for planning the expansion of healthcare and transportation infrastructure to improve geographic accessibility of cancer care. We present a new Facility Location Network Design Problem (FLNDP) in which a central planner can make improvements to existing healthcare facilities and to the existing road network over a multi-period planning horizon by allocating monetary and staffing budgets. During each period, some uncertainty is realized that impacts the demand for healthcare services from population centers and the travel time across the road network.

To solve this model, we formulate a new path-based mixed integer program (MIP). Our formulation has an exponentially large number of decision variables, which represent the possible paths from population centers to facilities in the network. To handle the scale of the problem, we first derive a branch-and-price algorithm. Leveraging the structure of the problem, we are able to efficiently solve the pricing problems using shortest-path subroutines. We further enhance the scalability of the algorithm by deriving acceleration techniques based on tailored heuristics, branching rules, and valid inequalities. Our final proposed algorithm is an enhanced branch-price-and-cut (BP&C) algorithm.

We test the algorithm on synthetic instances and observe that our acceleration techniques increase the rate at which optimal solutions are found on small instances and the likelihood that an optimal solution is found for larger instances. Further, we compare our algorithm to a commonly used variable neighbor search (VNS) heuristic from the existing literature. We find that VNS quickly provides high-quality solutions on small instances, but does not provide high-quality solutions as the instance size increases.

Finally, we apply our method to explore potential ways to expand access to tertiary cancer care in Rwanda over a five-year planning horizon. To do so, we create a high-fidelity network representation of the road network in Rwanda using Open Street Maps [15] and gather data on cancer care services from the Rwanda Ministry of Health and Partners in Health, a non-governmental organization. We also consider the impacts of oncologist scarcity and weather events in our planning.

In a representative scenario, our results reveal that cancer care is, on average, 30 minutes closer when investments in both healthcare and transportation infrastructure are considered, compared to improvements in healthcare infrastructure alone.

Moreover, our findings suggest that the recommendations from our FLNDP model could result in a 4-hour reduction in travel time to care for the patients who currently must travel the longest to access care, with one hour of this reduction being directly attributable to road network improvements. Our results demonstrate the value of jointly considering both network improvements and facility expansions, providing managerial insights to healthcare planners seeking to improve care access in LICs.

### Background and related literature

With the growing interest in understanding and addressing barriers to geographic access to care, modeling is starting to emerge as a powerful tool to help inform policy planning in LICs. While there has been a dearth of studies examining geographic access in LICs historically, recently there has been an emergence of the use of Geographical Information Systems (GIS) modeling to understand current levels of geographic access and how plans to expand cancer care would change geographic access in Rwanda [7], Ghana [16], and Nigeria [6].

While GIS modeling can be helpful to examine current levels of access and evaluate different expansion policies, these methods are limited in that they can only evaluate pre-defined plans, and there is no guarantee that these plans would make the best use of available resources. *Facility location modeling* extends these GIS approaches by using mathematical optimization to select the best facilities to improve a pre-specified measure of geographic access given limited resources. Despite its utility, very few studies have used facility location modeling for planning the expansion of cancer care services in LICs. Among those few studies are Sapirstein et al. [8] who examine the expansion of tertiary cancer care in Rwanda and de Campos et al. [17] who study the optimal location of mammography units in Brazil [17]. However, these existing studies do not consider improvements to the road network as a means to improve access to cancer care.

The operations research community has developed models and methods devoted to solving problems in which a planner must simultaneously choose where to locate facilities that can provide a service of interest and a transportation network that can be used to route demand to the selected location. Daskin et al. [18] provided the first formulation and coined such problems as FLNDPs. Shortly thereafter, Melkote and Daskin [19] demonstrated that an FLNDP can be reduced to a pure network design problem. Since then, considerable progress has been made on the ability to solve FLNDPs [20–23], which has enabled their use in various application areas, including healthcare.

Our work adds to a relatively recent stream of literature focusing on using FLNDPs to improve healthcare access in LICs. To the best of our knowledge, Cocking et al. [24] were the first to propose an FLNDP approach to improve access to healthcare through both facility expansions and road network improvements. They explore a single-period planning problem in which a decision-maker can build healthcare facilities and new roads to improve access to healthcare. They demonstrate their approach on an instance of an FLNDP representing Burkina Faso’s Nouna District, which they can solve using a commercial solver in a matter of hours. Ghaderi and Jabalameli [25] use an FLNDP approach to simultaneously design the capacity of healthcare facilities and the transfer network among these healthcare facilities over multiple time periods. The authors propose a fix-and-optimize approach to heuristically solve large instances of their problem, such as their case study of Iran’s Ilam Province. In contrast to our proposed model, Ghaderi and Jabalameli [25]’s model considers distinct monetary budgets for the construction of facilities and the construction of the transfer network. [26] also use an FLNDP model with distinct network and facility budgets, adopting a heuristic approach and considering health-system capacity constraints. More recently, Pourrezaie-Khaligh et al. [27] proposed an improvement on the VNS heuristic techniques of Ghaderi [22] while explicitly incorporating equity in their optimization model. While these studies have demonstrated the benefits of FLNDP as a means to improve healthcare access, the existing literature in this area has been limited to the solution of relatively small instances with a commercial solver or the solution of more realistic large-scale instances using heuristic approaches that do not provide any guarantees on the optimality gap. Therefore, there is a need for new optimization methods that can solve larger-scale instances to optimality or provide guarantees on the optimality gap. Our solution approach based on BP&C seeks to fill this gap.

The methods proposed in this article build upon previous efforts to solve large-scale FLNDPs. More specifically, our work builds on two streams of research in large-scale network design problems related to “edge-based” formulations for FLNDPs and “path-based” formulations for pure network design problems. Edge-based formulations use binary variables to represent the decisions of whether or not to construct an edge, continuous variables to represent the amount of demand from a demand point flowing along a given edge, and constraints to ensure that demand flows from a demand point along only constructed edges to reach a facility. Pearce and Forbes [23] were one of the first to propose an exact method for solving large-scale instances of edge-based formulations of FLNDPs. They use a Benders decomposition approach, adapted from a similar approach originally for pure network design problems [28], in which they fix the binary decision variables corresponding to facility and edge construction in a main problem. Then, they solve a subproblem that can be disaggregated by demand point and time period. Similar methods have been used in a variety of FLNDP contexts including hazardous waste transport [29], urban planning [30], and traffic flow [21]. Such edge-based formulations offer nice benefits because they lend themselves to decomposition techniques, such as Benders decomposition, which can improve solution times on large-scale instances. However, these formulations oftentimes struggle to converge on large instances: They still require solving many (potentially challenging) subproblems, leading to potentially substantial optimality gaps on large instances.

Path-based formulations are becoming popular in other network design problems. In these formulations, a path typically corresponds to a set of edges along which demand flows from the originating demand point of the path to a facility that satisfies this demand, which is the end point of the path. These formulations typically require fewer constraints than edge-based formulations, but this comes at the cost of requiring an enormous number of decision variables. Thus, these path-based formulations tend to be mixed-integer programs with a huge number of decision variables. Accordingly, existing studies have leveraged branch-and-price (B&P) [31] as a way to solve these formulations of pure network design problems [32, 33]. As a result, path-based formulations have been effective for generating solutions to many complex logistics, routing, and scheduling problems [34, 35]. To our knowledge, the only work on path-based formulations for FLNDPs has been limited to Contreras et al. [36]. In their deterministic single-period setting, they use a min-max objective function to minimize the maximum travel time across their network and demonstrate that a path-based formulation results in improved solution times compared to edge-based formulations. However, their approach relies on the generation of valid inequalities that result from the need to only consider the longest path. Thus, these valid inequalities do not generalize to the objective function considered in our modeling framework in which we consider population-average travel time, accounting for multiple improvement decision periods as well as demand and travel time uncertainty.

Despite the use of path-based approaches to solve a variety of pure network design problems and the strong connections between FLNDPs and pure network design problems, to our knowledge, the use of path-based formulations for solving FLNDPs has been extremely limited. In this article, we seek to create a solution methodology for our proposed FLNDP that combines the structure of path-based formulations with decomposition techniques more frequently seen in edge-based formations.

### Organization of this paper

The remainder of this article is organized as follows. In §2, we define our problem and formulate a two-stage stochastic MIP that can be used to solve it. In §3, we propose a new BP&C framework for solving the previously formulated MIP. In §4, we show the efficacy of our approach via computational experiments. In §5, we present our motivating case study and discuss the important findings. Finally, in §6, we conclude and identify future avenues of research.

## Problem description

### Problem statement

We consider a health system planner seeking to improve geographic access to tertiary cancer care for the regional population. Given a budget allocated over multiple time periods, the planner aims to invest in both expanding cancer services in existing facilities and improving road infrastructure. The planner decides on a single policy, which details these investments across all time periods. Considering uncertainty in the demand for cancer services and the impact of weather on road travel times, the planner’s objective is to minimize the expected travel time for the population to access cancer care.

Formally, we consider a set $\mathcal {O}$ of population centers with patients in need of cancer care. We denote by $\mathcal {F}$ the set of candidate healthcare facilities that can provide such care with some additional investment from the health system planner. We assume that such facilities are uncapacitated, as we reason that any newly constructed cancer care facility would be constructed to be sufficiently large to handle incoming demand. Population centers and facilities are connected by a road network, which the planner seeks to improve. Road surfaces (e.g., dirt, gravel, paved) can be categorized into an ordered set $\mathcal {S}= \{1,\dots ,S\}$ of S types, such that surface type $s \in \mathcal {S}$ is of lower quality than surface type $s^\prime \in \mathcal {S}$ when $s<s^\prime$. Next, we define as $\mathcal {G}:=(\mathcal {N},\mathcal {A})$ the undirected multigraph representing the potential improved road networks achievable after investment. Its set of nodes $\mathcal {N}$ comprises population centers, facilities, and road junctions, which are connected by a set of type-dependent arcs $\mathcal {A}$. Each arc $a = (i,j,s) \in \mathcal {A}$ reflects a potential road between nodes $i \in \mathcal {N}$ and $j \in \mathcal {N}$ with a surface of type $s \in \mathcal {S}$. Without loss of generality, we assume that each arc is associated with a single surface type; otherwise, a road can be equivalently represented by multiple smaller arcs of potentially different types.

At the beginning of a planning horizon, discretized into a set $\mathcal {T}:= \{1,\dots ,T\}$ of T (monthly/yearly) time periods, the planner selects a non-adaptive policy to be implemented across the planning horizon. Then, during each time period, $\tau \in \mathcal {T}$, the planner makes investments in the addition of cancer services to candidate healthcare facilities and in the improvement of the road network, according to the selected policy. Adding cancer services to a facility $f\in \mathcal {F}$ at the start of time period $\tau \in \mathcal {T}$ incurs both a one-time monetary cost $c^{\tau }_{f}$ and a human capital cost $h^{\tau }_{f}$, while improving the road between nodes *i* and *j* from surface type s-1 to type *s* incurs a one-time monetary cost $c^\tau _{ijs}$. We assume that road improvement costs are additive, namely, the cost of improving the road between *i* and *j* from surface type $s_1$ to type $s_2 > s_1$ at the beginning of time period τ is given by $\sum _{s=s_1+1}^{s_2} c^\tau _{ijs}$. We made this modeling choice to reflect the reality that dirt roads must be straightened and graveled prior to being paved. All costs, including maintenance and operational, associated with improving a road (resp. facility) in perpetuity can be included in $c_{ijs}^{\tau }$ (resp $c^{\tau }_{f}$). Further, we assume cancer services and road surfaces invested in at the beginning of a time period are available for the population during that time period. In each time period $\tau \in \mathcal {T}$, the planner receives a monetary budget $C^\tau$ and a staffing budget $H^{\tau }$. Any leftover budget that is not used by the end of a time period can be rolled over for use in the subsequent time period.

After the planner has made investments in a particular time period, the patients seeking cancer care travel across the road network to reach facilities offering cancer services. However, the planner faces uncertainty regarding the demand for such services. Furthermore, the condition of some roads (e.g., gravel or dirt) may significantly worsen during inclement weather (e.g., extreme rainfall), negatively impacting patients’ travel times. Thus, we consider a set Ω of stochastic scenarios that describe the uncertainty during all time periods: $\omega =(\omega _{1}, \omega _{2}, \ldots , \omega _{T})$.

Each scenario $\omega \in \Omega$ occurring with probability $p^{ \omega }$ is characterized by the demand $d^{\tau \omega }_{ o}$ for cancer services from each population center $o\in \mathcal {O}$, as well as the travel time $\ell ^{\tau \omega }_{a}$ across each type-dependent arc $a = (i,j,s) \in \mathcal {A}$ during each time period $\tau \in \mathcal {T}$. We naturally assume that in every scenario $\omega \in \Omega$ and time period $\tau \in \mathcal {T}$, arcs connecting two nodes *i* and *j* in the graph $\mathcal {G}$ satisfy $\ell ^{\tau \omega }_{ijs_1} > \ell ^{\tau \omega }_{ijs_2}$ for every $s_1 < s_2 \in \mathcal {S}$, i.e., the travel time to traverse a road of a given length will be higher if it is a lower-quality surface type than if it is a higher-quality surface type. We note that Ω can also account for additional factors such as potential armed conflicts or the effectiveness of cancer screening programs that can influence the likelihood of patients seeking care.

During each scenario and each time period, we assume that patients can accurately estimate the road network’s travel times, and select a path (i.e., route) that minimizes their travel time to a facility that provides cancer services. We define a path $\lambda \in \Lambda$ as a subset of adjacent type-dependent arcs in $\mathcal {A}$ that connect a population center $o_\lambda \in \mathcal {O}$ to a facility $f_\lambda \in \mathcal {F}$. Under the realized scenario $\omega \in \Omega$ during a time period $\tau \in \mathcal {T}$, the total travel time along each path $\lambda \in \Lambda$ is given by $\ell ^{\tau \omega }_{\lambda }:=\sum _{a \in \lambda } \ell ^{\tau \omega }_{a}$.

We then model the health system planner’s decisions as a two-stage stochastic multi-period facility location network design problem with uncapacitated facilities and uncapacitated arcs. In the first stage, the planner selects for each time period the subset of facilities to invest in the addition of cancer services and the subset of type-dependent arcs to construct, given the monetary and staffing budgets. Then, in the second stage, for a given realized scenario $\omega \in \Omega$ during each time period $\tau \in \mathcal {T}$, the patients from each population center $o\in \mathcal {O}$ select a shortest path $\hat{\lambda }^{\tau \omega }_o$ that crosses arcs that are constructed by time period τ to seek care in a facility that received investments by that same time period. Thus, the planner’s objective is to minimize the population’s expected travel time, given by

$$\begin{aligned} \sum _{\omega \in \Omega } p^{ \omega } {\sum _{\tau \in \mathcal {T}}} \left( \sum _{o\in \mathcal {O}} d^{\tau \omega }_{ o} \ell ^{\tau \omega }_{\hat{\lambda }^{\tau \omega }_{ o}}\right) . \end{aligned}$$

**Fig. 1:**
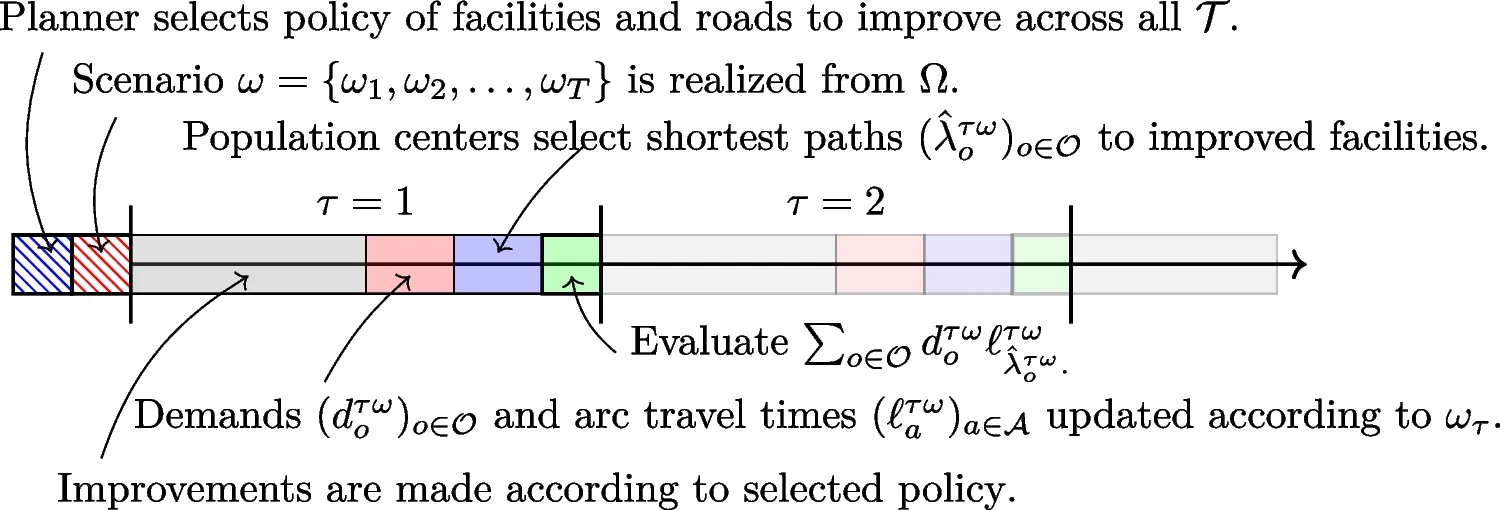
Decision and uncertainty timeline of the two-stage stochastic multi-period facility location network design problem. Policy is selected at the beginning of the horizon, but improvements are realized throughout the planning horizon

We illustrate the decision and uncertainty timeline in Fig. 1 and summarize the model notations in Table 1. Note that we interchangeably use the subscripts ijs and *a* when expressing parameters and variables that depend on a type-dependent arc $a = (i,j,s) \in \mathcal {A}$.

### Model formulation

We formulate the stochastic facility location network design problem described above as a mixed-integer program (MIP). The planner’s first-stage decisions can be described using four sets of binary decision variables: For each time period $\tau \in \mathcal {T}$, we use $u^\tau _f$ to indicate if the planner adds cancer services to facility $f\in \mathcal {F}$ at the start of time period τ. We also use $x^\tau _f$ to indicate if services are available at facility f at the start of time period τ, including the investment decision taken at the start of that time period. Similarly, $v^\tau _{a}$ (resp. $y^\tau _a$) determines if arc $a \in \mathcal {A}$ is constructed (resp. is available) at the start of time period τ. For each stochastic scenario $\omega \in \Omega$ that is realized, we define for every time period $\tau \in \mathcal {T}$ and path $\lambda \in \Lambda$ the second-stage continuous variable $z^{\tau \omega }_\lambda \in [0,1]$ that represents the fraction of demand from population center $o_\lambda$ (i.e., the origin of the path) traversing along λ to reach facility $f_\lambda$ (i.e., the end of the path).

We formulate the problem in Eq. (1) and summarize the decision variables in Table 2.

1a $$\begin{aligned}&\underset{u,v,x,y,z}{\text {minimize} }\sum _{\omega \in \Omega } p^{ \omega }\sum _{\tau \in \mathcal {T}} \sum _{\lambda \in \Lambda }d^{\tau \omega }_{ o_{\lambda }}\ell ^{\tau \omega }_{\lambda } z^{\tau \omega }_{ \lambda } \end{aligned}$$

1b $$\begin{aligned}&\text {subject to }{\sum _{\tau =1}^{\tau ^{\prime }}\left( \sum _{f\in \mathcal {F}} c^{\tau }_{f}u^{\tau }_{f} + \sum _{a \in \mathcal {A}} c^{\tau }_{a} v^{\tau }_{a}\right) }{\le \sum _{\tau =1}^{\tau ^{\prime }}C^{\tau }, \quad \forall \ \tau ^{\prime } \in \mathcal {T},} \end{aligned}$$

1c $$\begin{aligned}&{\sum _{\tau =1}^{\tau ^{\prime }}\sum _{f\in \mathcal {F}} h^{\tau }_{f}u^{\tau }_{f} }{\le \sum _{\tau =1}^{\tau ^{\prime }}H^{\tau }, \quad \forall \ \tau ^{\prime } \in \mathcal {T} ,} \end{aligned}$$

1d $$\begin{aligned}&{x^{\tau }_{f} - x^{\tau -1}_{f}}{= u^{\tau }_{ f}, \quad \forall \ f\in \mathcal {F}, \ \forall \ \tau \in \left\{ 2,\dots , T\right\} ,} \end{aligned}$$

1e $$\begin{aligned}&{y^{\tau }_{a} - y^{\tau -1}_{a}}{ = v^{\tau }_{a},\quad \forall \ a \in \mathcal {A}, \ \forall \ \tau \in \left\{ 2,\dots ,T\right\} ,} \end{aligned}$$

1f $$\begin{aligned}&{x^{1}_f}{= u^1_{f}, \quad \forall \ f\in \mathcal {F},} \end{aligned}$$

1g $$\begin{aligned}&{y^{1}_{a}}{= v^{1}_{a}, \quad \forall \ a \in \mathcal {A},} \end{aligned}$$

1h $$\begin{aligned}&{y^{\tau }_{ijs}}{\le y^{\tau }_{ij(s-1)}, \quad \forall \ (i,j,s) \in \mathcal {A}\, \text { s.t. }s \ge 2, \ \forall \ \tau \in \mathcal {T},} \end{aligned}$$

1i $$\begin{aligned}&{\sum _{\{\lambda \in \Lambda \, :\, o_{\lambda } = o\}}z^{\tau \omega }_{ \lambda }}{= 1 ,\quad \forall \ o\in \mathcal {O}, \ \forall \ \omega \in {\Omega }, \ \forall \ \tau \in \mathcal {T} ,} \end{aligned}$$

1j $$\begin{aligned}&{\sum _{\{\lambda \in \Lambda \, :\, o_\lambda = o, \, a \in \lambda \}}\hspace{-0.7cm}z^{\tau \omega }_{ \lambda }}{\le y^\tau _a, \quad \forall \ a \in \mathcal {A}, \ \forall \ o\in \mathcal {O}, \ \forall \ \omega \in {\Omega }, \ \forall \ \tau \in \mathcal {T},} \end{aligned}$$

1k $$\begin{aligned}&{\sum _{\{\lambda \in \Lambda \, :\, o_\lambda = o, \, f_\lambda = f\}}\hspace{-0.7cm}z^{\tau \omega }_{ \lambda } \le x^{\tau }_{f}, \quad \forall \ f\in \mathcal {F}, \ \forall \ o\in \mathcal {O}, \ \forall \ \omega \in {\Omega }, \ \forall \ \tau \in \mathcal {T} ,} \end{aligned}$$

1l $$\begin{aligned}&{z^{\tau \omega }_{ \lambda }}{\ge 0, \quad \forall \ \lambda \in \Lambda , \ \forall \ \omega \in {\Omega }, \ \forall \ \tau \in \mathcal {T},} \end{aligned}$$

1m $$\begin{aligned}&{x^{\tau }_{f}, \ u^{\tau }_{f} \in \{0,1\}}{,\quad \forall \ f\in \mathcal {F}, \ \forall \ \tau \in \mathcal {T} ,} \end{aligned}$$

1n $$\begin{aligned}&{y^{\tau }_{a}, \ v^{\tau }_{a} \in \left\{ 0,1\right\} }{,\quad \forall \ a \in \mathcal {A},\ \forall \ \tau \in \mathcal {T}.} \end{aligned}$$

**Table 1:** Notation used to describe the FLNDP

Symbol	Description
$\mathcal {N}$	Set of network nodes
$\mathcal {F}$	Set of facility locations, $\mathcal {F}\subseteq \mathcal {N}$
$\mathcal {O}$	Set of population centers, $\mathcal {O}\subseteq \mathcal {N}$
$\mathcal {S}$	Set of road surface types, $\mathcal {S}= \left\{ 1,\dots , S\right\}$
$\mathcal {A}$	Set of type-dependent arcs
$\mathcal {G}$	Graph of potential improved road networks, $\mathcal {G}= (\mathcal {N},\mathcal {A})$
Λ	Set of paths in graph $\mathcal {G}$ connecting a population center to a facility
$\mathcal {T}$	Set of time periods, $\mathcal {T} = \left\{ 1,\dots , T\right\}$
Ω	Set of stochastic scenarios
$c^{\tau }_{ijs}$	Monetary cost to construct arc $(i,j,s) \in \mathcal {A}$ at the start of period $\tau \in \mathcal {T}$
$c^{\tau }_{f}$	Monetary cost of adding services to facility $f\in \mathcal {F}$ at the start of period $\tau \in \mathcal {T}$
$h^{\tau }_{f}$	Human capital cost of adding services to $f\in \mathcal {F}$ at the start of period $\tau \in \mathcal {T}$
$C^{\tau }$	Monetary budget received at the start of period $\tau \in \mathcal {T}$
$H^\tau$	Staffing budget received at the start of period $\tau \in \mathcal {T}$
$o_\lambda$	Population center from where path $\lambda \in \Lambda$ originates
$f_\lambda$	Facility at which path $\lambda \in \Lambda$ terminates
$p^{ \omega }$	Probability of scenario $\omega \in \Omega$
$d^{\tau \omega }_{ o}$	Demand at population center $o \hspace{-0.035cm}\in \hspace{-0.035cm} \mathcal {O}$ during period $\tau \hspace{-0.035cm} \in \hspace{-0.035cm} \mathcal {T}$ under scenario $\omega \hspace{-0.035cm} \in \hspace{-0.035cm} \Omega$
$\ell ^{\tau \omega }_{a}$	Travel time along arc $a \in \mathcal {A}$ during period $\tau \in \mathcal {T}$ under scenario $\omega \in \Omega$
$\ell ^{\tau \omega }_{\lambda }$	Travel time along path $\lambda \in \Lambda$ during period $\tau \in \mathcal {T}$ under scenario $\omega \in \Omega$

The objective function (1a) computes the cumulative expected travel time over the entire planning horizon. Constraints (1b-1c) ensure that at each time period, the planner does not exceed the cumulative monetary and staffing budgets received by then. The linking constraints (1d-1g) guarantee that the variables *x* and *y* respectively represent the availability of facilities and arcs at the start of each time period, as a result of the investment decision variables *u* and *v*. By enforcing that an arc of type *s* can only be constructed if lesser arc types have also been constructed, constraints (1h) model the additive nature of road improvement costs. Constraints (1i) account for the fact that all patients seeking care in each scenario travel to a facility. Constraints (1j-1k) ensure that in each possible scenario and each time period, any path selected by a patient only traverses available type-dependent arcs to reach a facility with available services. We note that constraints (1d-1g) can be adjusted to account for construction lead times. Furthermore, monetary and human capital costs can be set to zero for any facility that already provides services. Similarly, if an arc $(i,j,s) \in \mathcal {A}$ is already constructed, then we set $c_{ijs^\prime }^\tau = 0$ for every time period τ and every type $s^\prime \in \{1,\dots ,s\}$. Finally, we observe that at optimality of the MIP (), every patient will select in each scenario a shortest path that traverses improved roads to reach a facility with available services.

**Table 2:** Decision variables used in the MIP formulation of the two-stage stochastic multi-period FLNDP

Variable	Description
$u^{\tau }_{f}\in \left\{ 0,1\right\}$	1 if services are added to facility $f\in \mathcal {F}$ at the start of period $\tau \in \mathcal {T}$
$v^{\tau }_{a}\in \left\{ 0,1\right\}$	1 if arc $a \in \mathcal {A}$ is constructed at the start of period $\tau \in \mathcal {T}$
$x^{\tau }_{f}\in \left\{ 0,1\right\}$	1 if services are available at facility $f\in \mathcal {F}$ at the start of period $\tau \in \mathcal {T}$
$y^{\tau }_{a}\in \left\{ 0,1\right\}$	1 if arc $a \in \mathcal {A}$ is available at the start of period $\tau \in \mathcal {T}$
$z^{\tau \omega }_{\lambda }\in [0,1]$	Fraction of demand from population center $o_\lambda \in \mathcal {O}$ using path $\lambda \in \Lambda$ in scenario $\omega \in \Omega$ during time period $\tau \in \mathcal {T}$

The resulting formulation requires $2T(|\mathcal {F}| + |\mathcal {A}|)$ binary first-stage decision variables and $T|\Omega ||\Lambda |$ continuous second-stage variables. Since the number of paths is exponential, () cannot directly be solved using commercial solvers and requires new methodologies based on decomposition algorithms.

## Solution methodology

### Algorithm overview

In this section, we first develop an algorithm based on B&P for solving the large-scale MIP (). This tailored algorithm iteratively solves the linear programming (LP) relaxation of () using column generation and recursively splits the search space using a branch-and-bound algorithm to find the best integer-feasible solution.

To solve the LP relaxation of (), referred to as the master problem, we iteratively compute an optimal solution to a restricted version that considers all first-stage binary variables, but only a small subset of second-stage path variables. We then solve the pricing subproblem to determine the variable in the original master problem with the lowest reduced cost. If the lowest reduced cost is negative, the corresponding variable is added to the restricted master problem, which is subsequently solved. Column generation terminates when all reduced costs are nonnegative, guaranteeing that the optimal solution to the restricted master problem is optimal for the original master problem.

If the resulting solution to the master problem satisfies the integrality constraints (1m-1n), then the branch-and-price algorithm terminates with an optimal solution to (). Otherwise, the algorithm branches on a first-stage variable with a fractional value, creating new problems to be solved using column generation. Each problem is represented as a node of a branch-and-bound tree, and the algorithm terminates when all tree nodes have been processed. The algorithm updates the incumbent solution as it obtains integer-feasible solutions with lower objective values. At optimality, the incumbent solution is optimal for (). We note that if the LP relaxation at a given tree node has a fractional optimal solution with an objective value that is no less than that of the incumbent solution, then the algorithm does not branch on that fractional solution, as it cannot lead to a better incumbent solution. Furthermore, column generation at each tree node is warm-started using all columns that have been generated at previously processed nodes.

To improve scalability of the algorithm we leverage the problem’s structure to develop acceleration techniques. The first innovative element of our approach consists of designing a heuristic procedure to initialize the B&P algorithm with a good-quality incumbent solution, leading to a smaller number of problems to solve. The heuristic first solves a facility location model without considering any road improvements and then solves another problem that uses the remaining budget to improve roads. In the second acceleration technique, we leverage the multi-period structure to adjust the branching rule using special ordered sets. Finally, we develop valid inequalities based on optimal dual solutions of the second-stage problem to reduce the number of iterations of column generation in subsequent tree nodes. We add these valid inequalities when the algorithm finds an integer-feasible solution, making our tailored approach a BP&C algorithm.

### Column generation

We solve every LP relaxation at a node of the branch-and-bound tree using the column generation algorithm. Given a tree node, let $\mathcal {B}$ denote the set of constraints obtained by branching on first-stage variables at its ancestor nodes. For instance, $\mathcal {B}$ may enforce services to be added to a subset of facilities at the start of some periods or require services to be unavailable at the start of some periods. Similarly, this set encapsulates constraints that require arcs to be available at the start of some periods, or that prevent arcs from being constructed at the start of some periods. The master problem we aim to solve at that tree node can be formulated as the following linear program:

2a $$\begin{aligned} \mathcal {L}(\mathcal {B},\Lambda ): \underset{u,v,x,y,z}{\text {minimize}}&{\sum _{\omega \in \Omega } p^{ \omega }\sum _{\tau \in \mathcal {T}}\sum _{\lambda \in \Lambda }d^{\tau \omega }_{ o_{\lambda }}\ell ^{\tau \omega }_{\lambda } z^{\tau \omega }_{ \lambda } } \end{aligned}$$

2b $$\begin{aligned} \text {subject to}&~{\text {Eqs. (1b-1k)}}{,}\nonumber \\&{z^{\tau \omega }_{ \lambda }}{\ge 0,}{\quad \forall \ \lambda \!\in \! \Lambda , \ \forall \ {\omega \!\in \! \Omega }, \ \forall \ \tau \in \mathcal {T},} \end{aligned}$$

2c $$\begin{aligned}&{0 \le x^{\tau }_{f}, \ u^{\tau }_{f}}{ \le 1,}{\quad \forall \ f\in \mathcal {F}, \ \forall \ \tau \in \mathcal {T} ,}\end{aligned}$$

2d $$\begin{aligned}&{0\le y^{\tau }_{a}, \ v^{\tau }_{a}}{\le 1,}{\quad \forall \ a \in \mathcal {A},\ \forall \ \tau \in \mathcal {T},}\end{aligned}$$

2e $$\begin{aligned}&{(u,v,x,y) \in \mathcal {B}}{.} \end{aligned}$$

The master problem $\mathcal {L}(\mathcal {B},\Lambda )$ cannot be solved directly using commercial solvers due to the extremely large number of path variables. Instead, we develop a column generation algorithm that iteratively introduces subsets of variables. Specifically, we solve the restricted master problem $\mathcal {L}(\mathcal {B},\bar{\Lambda })$, obtained by only considering a subset of the path variables $\bar{\Lambda } \subseteq \Lambda$. We denote by $(\hat{u},\hat{v},\hat{x},\hat{y},\hat{z})$ the resulting optimal solution to the restricted master problem. To determine whether or not this solution is optimal for the original master problem $\mathcal {L}(\mathcal {B},\Lambda )$, we check whether any excluded path variable $z_{\lambda }^{\tau \omega }$ for $\lambda \in \Lambda \setminus \bar{\Lambda }$ has a negative reduced cost.

For every $o\in \mathcal {O}$, $f \in \mathcal {F}$, $a \in \mathcal {A}$, $\tau \in \mathcal {T}$, and $\omega \in \Omega$, we let $\hat{\alpha }_o^{\tau \omega }$, $\hat{\beta }_{oa}^{\tau \omega }$, and $\hat{\gamma }_{of}^{\tau \omega }$ respectively represent the dual variables associated with Eqs. (1i), (1j), and (1k) at optimality of $\mathcal {L}(\mathcal {B},\bar{\Lambda })$. Then, the reduced cost of *any* path variable $z_{\lambda }^{\tau \omega }$ is given by:

3 $$\begin{aligned} \overline{c}(z_{\lambda }^{\tau \omega }):= &   {p^{ \omega }}d^{\tau \omega }_{ o_\lambda }\ell ^{\tau \omega }_{ \lambda } -\hat{\alpha }^{\tau \omega }_{ o_{\lambda }} - \sum _{i,j,s\in \lambda } \hat{\beta }^{\tau \omega }_{o_\lambda a}- \hat{\gamma }_{o_\lambda f_\lambda }^{\tau \omega }, \nonumber \\  &   \forall \ \lambda \in \Lambda , \ \forall \ {\omega \in \Omega }, \ \forall \ \tau \in \mathcal {T}. \end{aligned}$$

To avoid complete enumeration of the exponentially large number of such reduced costs, we instead formulate the pricing problem, which aims to find the variable with the lowest reduced cost. It is critical to efficiently solve this pricing problem to enable scalability of the column generation algorithm. By leveraging the network structure of the problem, we show in the next proposition that the pricing problem can be solved in polynomial time using shortest-path subroutines:

#### Proposition 1

Given a population center $o\in \mathcal {O}$, a scenario $\omega \in \Omega$, and a time period $\tau \in \mathcal {T}$, the lowest reduced cost of a variable $z^{\tau \omega }_{ \lambda }$ corresponding to path λ that originates at location *o* is given by

4 $$\begin{aligned} \min _{\{\lambda \in \Lambda \, : \, o_\lambda = o\}}\sum _{a \in \lambda }\left( {p^{ \omega }}d^{\tau \omega }_{o} \ell ^{\tau \omega }_{a} - \hat{\beta }^{\tau \omega }_{oa}\right) - \hat{\alpha }^{\tau \omega }_{o} - \hat{\gamma }^{\tau \omega }_{of_\lambda }, \end{aligned}$$

which can be solved as a shortest-path problem in $O(|\mathcal {N}||\mathcal {A}|)$ time.

Therefore, computing the variable in $\mathcal {L}(\mathcal {B},\Lambda )$ with lowest reduced cost can be achieved in $O(|\mathcal {N}|^2|\mathcal {A}|\sum _{\tau \in \mathcal {T}} |\Omega ^\tau |)$ time.

#### Proof of Proposition 1

Let $o \in \mathcal {O}$, $\omega \in \Omega$, and $\tau \in \mathcal {T}$. We create a graph $\mathcal {G}^{\tau \omega }_{o}$ as a copy of the graph of potential improved road networks $\mathcal {G}= (\mathcal {N},\mathcal {A})$. Next, we augment $\mathcal {G}^{\tau \omega }_{o}$ by adding a dummy sink node $\bar{f}$ and connecting it to each facility $f \in \mathcal {F}$ with a single arc. The resulting graph is then given by $\mathcal {G}^{\tau \omega }_{o} = (\mathcal {N}\cup \{\bar{f}\}, \mathcal {A} \cup \{(f,\bar{f}), \ \forall \ f \in \mathcal {F}\})$. We set the length $l_a$ of each arc *a* in $\mathcal {G}^{\tau \omega }_{o}$ as follows:

5 $$\begin{aligned} l_{a} = \left\{ \begin{array}{lll} {p^{ \omega }}d^{\tau \omega }_{ o}\ell ^{\tau \omega }_{ a} - \hat{\beta }^{\tau \omega }_{oa}&  &  \text {if } a \in \mathcal {A}\\ - \hat{\alpha }^{\tau \omega }_{o} - \hat{\gamma }^{\tau \omega }_{of} &  & \text {if } a = (f,\bar{f}) \text { for some } f\in \mathcal {F}. \end{array}\right. \end{aligned}$$

An example of such a graph is provided in Appendix B.1. We note that all arcs $a \in \mathcal {A}$ have nonnegative lengths since $\hat{\beta }_{oa}^{\tau \omega } \le 0$.

In the graph $\mathcal {G}^{\tau \omega }_{o}$, we observe that the length of every path originating at *o* and traversing facility *f* to reach $\bar{f}$ has a length of $\sum _{a \in \lambda }\left( p^{ \omega }d^{\tau \omega }_{o} \ell ^{\tau \omega }_{ a} - \hat{\beta }^{\tau \omega }_{oa}\right) - \hat{\alpha }^{\tau \omega }_{o} - \hat{\gamma }^{\tau \omega }_{of}$. Therefore, Problem Eq. (4) can be solved by determining the shortest path in $\mathcal {G}^{\tau \omega }_{o}$ from *o* to $\bar{f}$. The Bellman-Ford algorithm finds such a path in $O((|\mathcal {N}|+1)(|\mathcal {A}|+|\mathcal {F}|)) = O(|\mathcal {N}||\mathcal {A}|)$ time.

By repeating this process for every $o \in \mathcal {O}$, $\tau \in \mathcal {T}$, and $\omega \in \Omega$, we can find the variable $z_{\lambda }^{\tau \omega }$ with the lowest reduced cost in $O(|\mathcal {N}|^2|\mathcal {A}||\mathcal {T}| |\Omega |)$ time.

From Proposition 1, we obtain that the pricing problem can be efficiently solved by running $|\mathcal {O}||\Omega ||\mathcal {T}|$ shortest-path algorithms. Let $\hat{\lambda }_o^{\tau \omega } \in \Lambda$ represent an optimal solution of Eq. (4) for every $o\in \mathcal {O}$, $\tau \in \mathcal {T}$, and $\omega \in \Omega$. If $\bar{c}(z^{\tau \omega }_{\hat{\lambda }_o^{\tau \omega }}) \ge 0$ for every $o\in \mathcal {O}$, $\tau \in \mathcal {T}$, and $\omega \in \Omega$, then $(\hat{u},\hat{v},\hat{x},\hat{y},\hat{z})$ is an optimal solution of the master problem $\mathcal {L}(\mathcal {B},\Lambda )$. Otherwise, we augment $\bar{\Lambda }$ by including the paths we have found with negative reduced costs and solve the new restricted master problem.

We note that our approach deviates from a traditional implementation of column generation in that if a path λ is identified such that $\bar{c}(z_{\lambda }^{\tau ^\prime \omega ^\prime }) < 0$ for some $\tau ^\prime \in \mathcal {T}$ and $\omega ^\prime \in {\Omega }$, then the corresponding variables $z_{\lambda }^{\tau \omega }$ are added to the new restricted master problem for *all* $\tau \in \mathcal {T}$ and $\omega \in \Omega$. Due to the structure of the problem, the same path is likely to be used by patients across many time periods and scenarios, thus adding all corresponding variables reduces the number of iterations of the column generation algorithm. We detail our implementation of column generation in Algorithm 1.

**Algorithm 1:**
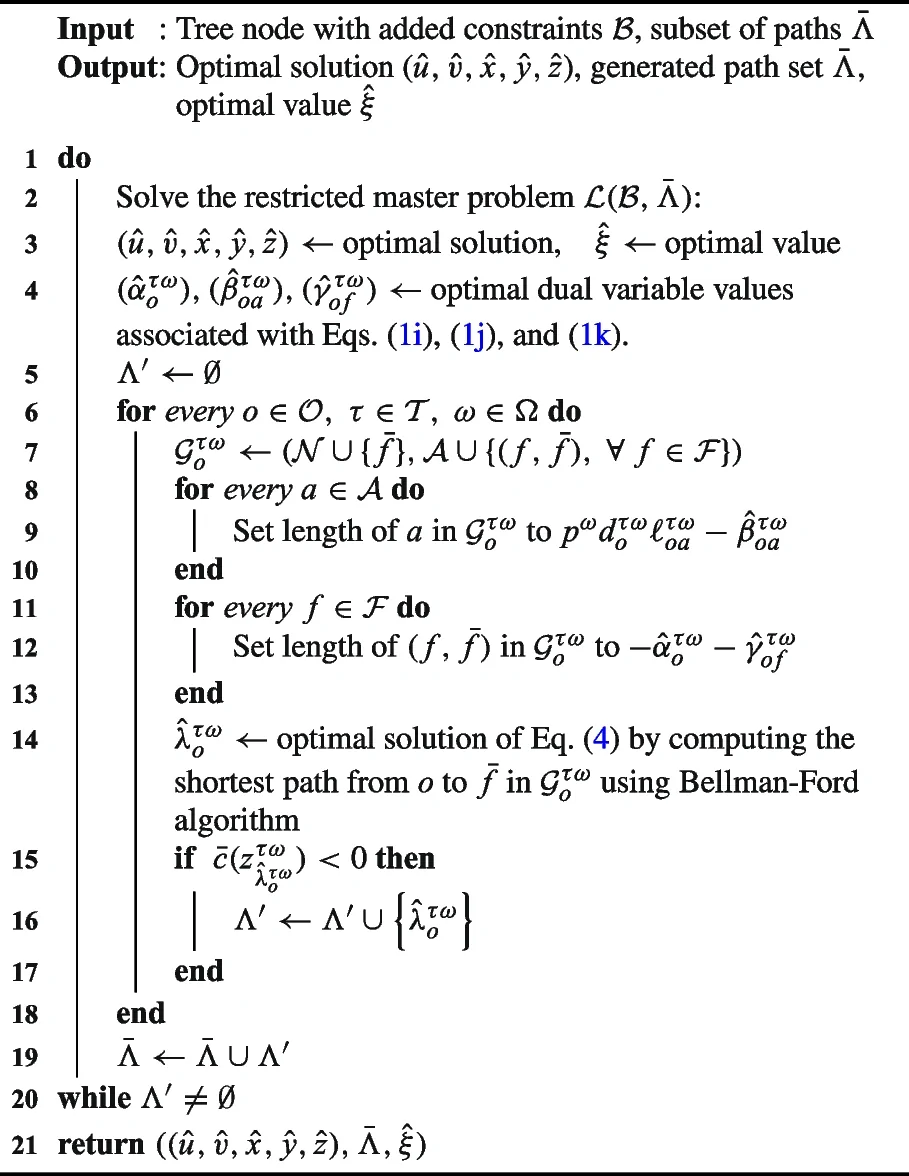
Column generation for solving $\mathcal {L}(\mathcal {B},\Lambda )$.

### Branch-and-price algorithm

We now formally describe in Algorithm 2 our custom branch-and-price algorithm for solving Eq. (1).

**Algorithm 2:**
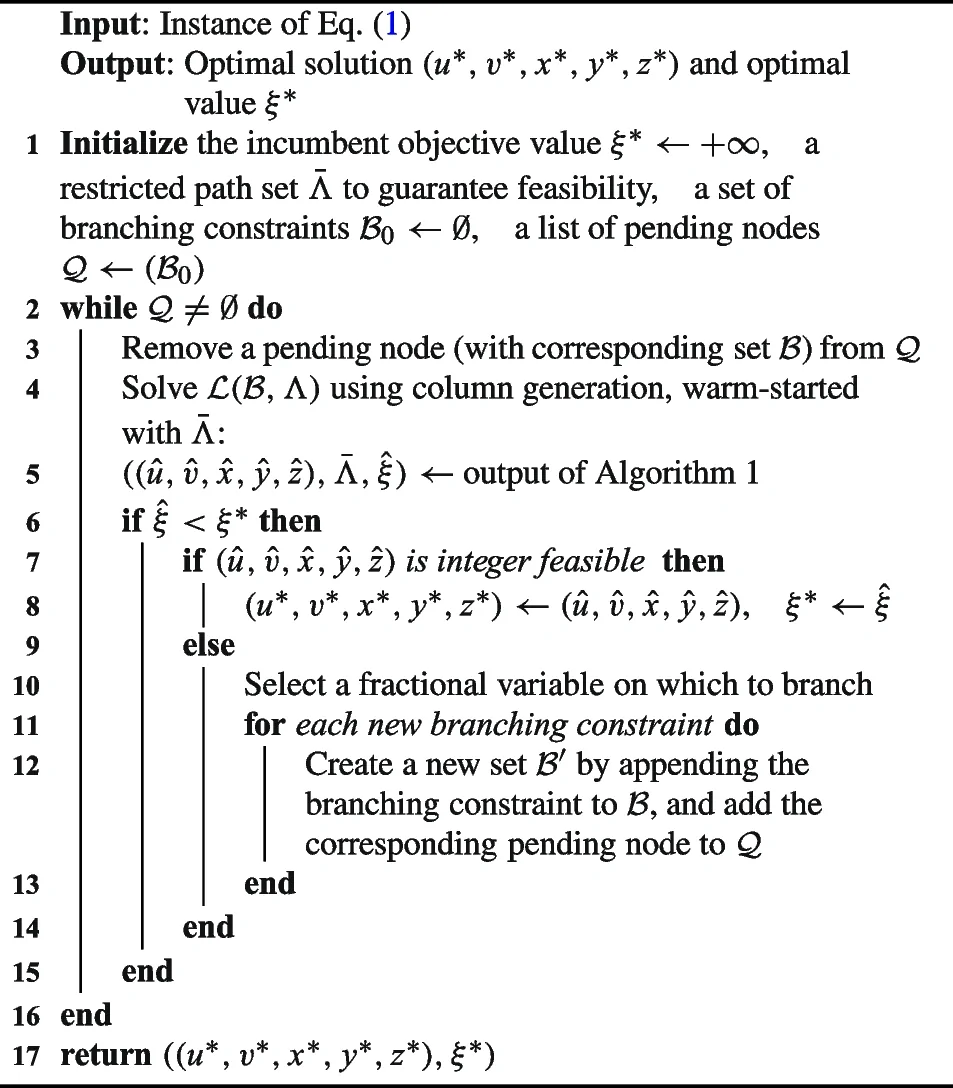
Branch-and-price algorithm for solving Eq. (1).

### Acceleration techniques

To further enhance the scalability of the B&P algorithm, we devise additional acceleration techniques that leverage the structure of the problem.

#### Heuristic solution

We design a two-step optimization-based method for generating good-quality feasible solutions of Eq. (), providing a strong incumbent solution at the onset of Algorithm 2. In this method, we first solve a simpler version of Eq. () by assuming that the planner can only invest in adding services to facilities. Then, we solve a pure network design problem that uses the budget that is left over after expanding facilities to improve the road network.

To solve the first problem, we consider a dummy sink node $\bar{f}$, and connect it to each facility $f \in \mathcal {F}$ with a dummy arc of length equal to zero. We also let $\mathcal {A}^0 \subseteq \mathcal {A} \cup \{(f,\bar{f}), \ \forall \ f \in \mathcal {F}\}$ denote the set of arcs already constructed in the road network (including the dummy arcs). For the sake of exposition, we assume that all arcs in $\mathcal {A}^0$ are of surface type 1. Then, we consider the same variables *u* (resp. *x*) to indicate when cancer services are added (resp. available) at each facility. However, we replace the second-stage decisions with continuous variables $w_{a}^{\tau \omega }$, representing the flow of patients (originating at any population center) traveling along arc $a \in \mathcal {A}^0$ during scenario $\omega \in \Omega$ at time period $\tau \in \mathcal {T}$. Since arcs in $\mathcal {A}^0$ are originally undirected, we arbitrarily direct them. Consequently, $w_{a}^{\tau \omega } < 0$ signifies that $-w_{a}^{\tau \omega }$ patients are traveling along arc *a* in its opposite direction. We also introduce auxiliary variables $\theta _a^{\tau \omega }$ (for $a \in \mathcal {A}$, $\omega \in \Omega$, $\tau \in \mathcal {T}$) to represent the absolute value of $w_{a}^{\tau \omega }$, which corresponds to the nonnegative number of patients traveling along arc *a*. For every node $i \in \mathcal {N}\cup \{\bar{f}\}$, we denote by $\delta ^+(i) \subseteq \mathcal {A}^0$ (resp. $\delta ^-(i) \subseteq \mathcal {A}^0$) its subset of outgoing arcs (resp. incoming arcs). The problem can then be formulated as the following MIP:

6a $$\begin{aligned} {\underset{u,x,w,\theta }{\text {minimize}}}&{\sum _{\omega \in \Omega } p^{ \omega }}\sum _{\tau \in \mathcal {T}}\sum _{a \in \mathcal {A}^0}\ell ^{\tau \omega }_{a} \theta ^{\tau \omega }_{a}   \end{aligned}$$

6b $$\begin{aligned} \text {subject to }&{\sum _{\tau = 1}^{\tau ^{\prime }} \sum _{f\in \mathcal {F}} c_{f}^{\tau } u^{\tau }_{f} \le \sum _{\tau = 1}^{\tau ^{\prime }} C^{\tau },}{\ \forall \ \tau ^\prime \in \mathcal {T},} \end{aligned}$$

6c $$\begin{aligned}&{\sum _{\tau = 1}^{\tau ^{\prime }} \sum _{f\in \mathcal {F}} h_{f}^{\tau } u^{\tau }_{f} \le \sum _{\tau = 1}^{\tau ^{\prime }} H^{\tau },}{\ \forall \ \tau ^\prime \in \mathcal {T},} \end{aligned}$$

6d $$\begin{aligned}&{x_f^\tau - x_f^{\tau - 1} = u_f^\tau ,}{\ \forall \ f\in \mathcal {F}, \ \forall \ \tau \in \{2,\dots ,T\},} \end{aligned}$$

6e $$\begin{aligned}&{x^1_{f} = u^{1}_{f},}{ \ \forall \ f\in \mathcal {F},} \end{aligned}$$

6f $$\begin{aligned}&{-\theta _a^{\tau \omega } \le w_a^{\tau \omega } \le \theta _a^{\tau \omega },}{ \ \forall \ a \in \mathcal {A}, \ \forall \ \omega \in {\Omega }, \ \forall \ \tau \in \mathcal {T},} \end{aligned}$$

6g $$\begin{aligned}&{\sum _{a \in \delta ^+(i)}w_{a}^{\tau \omega } - \sum _{a \in \delta ^-(i)}w_{a}^{\tau \omega } = \left\{ \begin{array}{ll} d_i^{\tau \omega } &  \text { if } i \in \mathcal {O}\\ \displaystyle -\sum _{o \in \mathcal {O}} d_o^{\tau \omega } &  \text { if } i=\bar{f}\\ 0 &  \text { otherwise},\end{array}\right. }\nonumber \\&\qquad \qquad \qquad \qquad \qquad \quad {\forall \ i \in \mathcal {N} \cup \{\bar{f}\}, \ \forall \ \omega \in {\Omega }, \ \forall \tau \in \mathcal {T}, } \end{aligned}$$

6h $$\begin{aligned}&{-\sum _{o \in \mathcal {O}} d_o^{\tau \omega } x_f^\tau \!\le \! w_{f\bar{f}}^{\tau \omega } \!\le \! \sum _{o \in \mathcal {O}} d_o^{\tau \omega } x_f^\tau ,}{\ \forall \ f \!\in \! \mathcal {F}, \ \forall \ \omega \!\in \! {\Omega }, \ \forall \tau \!\in \! \mathcal {T},} \end{aligned}$$

6i $$\begin{aligned}&{x^{\tau }_{f}, \ u^{\tau }_{f} \in \left\{ 0,1\right\} }{,\quad \forall \ f\in \mathcal {F}, \ \forall \ \tau \in \mathcal {T}.} \end{aligned}$$

In this MIP, the flow conservation constraints Eq. (6g) ensure that patients travel from their population centers to the dummy sink node (after going through one facility). Furthermore, constraints Eq. (6h) prevent patients from traveling to a facility where services are not available.

We solve the smaller MIP Eq. (6) directly with commercial solvers; we denote its optimal solution by $(\hat{u},\hat{x},\hat{w},\hat{\theta })$. In the next step, we allocate the remaining monetary budget to improve the road network: We develop a second optimization problem to minimize the patients’ expected travel times, assuming that they will travel according to $\hat{\theta }$ towards facilities with service availability governed by $\hat{x}_f^\tau$ at optimality of Eq. (). Given the same variables *v* and *y* to indicate when arcs are constructed and available, we formulate the following MIP:

7a $$\begin{aligned}  &   \underset{v,y}{\text {minimize}\,\,\,\,} {{\sum _{\omega \in \Omega } p^{ \omega }} \sum _{\tau \in \mathcal {T}} \sum _{(i,j,s) \in \mathcal {A}} \hat{\theta }_{ij1}^{\tau \omega } (\ell _{ijs}^{\tau \omega } - \ell _{ij(s-1)}^{\tau \omega }) y_{ijs}^{\tau }}  \end{aligned}$$

7b $$\begin{aligned}  &   \text {subject to } {\sum _{\tau =1}^{\tau ^{\prime }}\sum _{a \in \mathcal {A}} c^{\tau }_{a} v^{\tau }_{a}}{\le \sum _{\tau =1}^{\tau ^{\prime }}\left( C^{\tau } - \sum _{f\in \mathcal {F}} c^{\tau }_{f}\hat{u}^{\tau }_{f}\right) , \quad \forall \ \tau ^{\prime } \in \mathcal {T},} \end{aligned}$$

7c $$\begin{aligned}  &   {y^{\tau }_{a} - y^{\tau -1}_{a}}{ = v^{\tau }_{a},\quad \forall \ a \in \mathcal {A}, \ \forall \ \tau \in \left\{ 2,\dots ,T\right\} ,} \end{aligned}$$

7d $$\begin{aligned}  &   {y^{1}_{a}}{= v^{1}_{a}, \quad \forall \ a \in \mathcal {A},} \end{aligned}$$

7e $$\begin{aligned}  &   {y^{\tau }_{ijs}}{\le y^{\tau }_{ij(s-1)}, \quad \forall \ (i,j,s) \in \mathcal {A}\, \text { s.t. }s \ge 2, \ \forall \ \tau \in \mathcal {T},} \end{aligned}$$

7f $$\begin{aligned}  &   {y^{\tau }_{a}, \ v^{\tau }_{a} \in \left\{ 0,1\right\} }{,\quad \forall \ a \in \mathcal {A},\ \forall \ \tau \in \mathcal {T},} \end{aligned}$$

with the convention that $\ell _{ij0}^{\tau \omega } := 0$. We note that the inner summation in the objective function Eq. (7a) indeed represents the travel time incurred by the $\hat{\theta }_{ij1}^{\tau \omega }$ patients traveling between *i* and *j* on the best surface type available during time period τ.

We solve the small MIP Eq. (7) using commercial solvers and denote by $(\hat{v},\hat{y})$ (resp. $\hat{\xi }$) its optimal solution (resp. optimal value). From this approach, we obtain feasible first-stage variables $\hat{u},\hat{v}, \hat{x}, \hat{y}$ of Eq. () and an incumbent objective value to warm-start our branch-and-price algorithm.

#### Branching rules

Conventional bifurcated branching in branch-and-bound divides the search space at a given node into two subproblems by enforcing that a fractional variable at the node takes on a value of either 0 or 1. These subproblems are added as child nodes in the tree. For instance, if column generation outputs an optimal solution to the LP relaxation $\mathcal {L}(\mathcal {B},\Lambda )$ at a tree node with a fractional first-stage variable $\hat{u}_f^\tau$ for some $f \in \mathcal {F}$ and $\tau \in \mathcal {T}$, then conventional branching will create two child nodes, where in the first (resp. second) node, the constraint $u_f^\tau = 0$ (resp. $u_f^\tau = 1$) is added to $\mathcal {B}$.

From our problem’s structure, there is at most one period at the start of which cancer services are added to a facility (or similarly, an arc is constructed). Thus, the improvement decision variables *u* and *v* form a special ordered set with at most one non-zero entry. We use this structure in our branching strategy. For instance, if $\hat{u}_f^\tau$ is fractional at optimality of $\mathcal {L}(\mathcal {B},\Lambda )$ at a tree node for some $f \in \mathcal {F}$ and $\tau \in \mathcal {T}$, we implement this special wide branching as follows. Instead of branching on the $u_f^\tau$ variables corresponding to facility f separately, we branch on the set $(u_f^1,\ldots ,u_f^T)$ to identify when (if ever) facility f will be expanded. This process creates T+1 subproblems, one for each $\tau \in \mathcal {T}$ to represent that $u_f^\tau = 1$ and $u_f^{\tau '}=0$ for $\tau '\ne \tau$, and a final subproblem corresponding to $u_f^\tau =0,\;\forall \ \tau \in \mathcal {T}$. The process is similar for arc construction variables *v*.

Additionally, sequential decisions by the planner imply that fixing first-stage decisions in earlier periods reduces the number of feasible decisions in later periods. Therefore, we prioritize branching on fractional variables in earlier periods before considering those in later periods. We found that this branching strategy accelerates the convergence of our algorithm.

#### Valid inequalities

As we process nodes of the branch-and-bound tree, we aim to incorporate information from the optimal solutions of the associated LP relaxations into the remaining tree nodes. To this end, we investigate valid inequalities, as described in the following proposition:

##### Proposition 2

Given a population center $o\in \mathcal {O}$, a time period $\tau \in \mathcal {T}$ and a scenario $\omega \in \Omega$, valid inequalities to the MIP Eq. () are given by

8 $$\begin{aligned} \sum _{\{\lambda \!\in \! \Lambda \, :\, o_\lambda = o\}} \ell ^{\tau \omega }_{ \lambda }z^{\tau \omega }_{ \lambda } \!\ge \! \alpha ^{\tau \omega }_{o} \!+\! \sum _{a \in \mathcal {A}} \beta ^{\tau \omega }_{oa} x^{\tau }_{a} \!+\! \sum _{f\in \mathcal {F}} \gamma ^{\tau \omega }_{of} w^{\tau }_{f} , \end{aligned}$$

for any $\alpha ,\beta ,\gamma$ satisfying

9 $$\begin{aligned} \alpha _o^{\tau \omega } + \sum _{a \in \lambda }\beta _{oa}^{\tau \omega }+ \gamma _{of_\lambda }^{\tau \omega } \le \ell ^{\tau \omega }_{\lambda },&\quad \forall \ \lambda \in \Lambda \ :\ o_\lambda = o,\end{aligned}$$

10 $$\begin{aligned} \beta _{oa}^{\tau \omega } \le 0,&\quad \forall \ a \in \mathcal {A},\end{aligned}$$

11 $$\begin{aligned} \gamma _{of}^{\tau \omega } \le 0,&\quad \forall \ f \in \mathcal {F}. \end{aligned}$$

##### Proof of Proposition 2

We consider a feasible solution $(\hat{u},\hat{v}, \hat{x}, \hat{y}, \hat{z})$ to Eq. (), a population center $o\in \mathcal {O}$, a time period $\tau \in \mathcal {T}$, and a scenario $\omega \in \Omega$. By definition of Eq. (), we obtain that:

$$\begin{aligned} \sum _{\{\lambda \in \Lambda \, :\, o_\lambda = o\}} \ell _\lambda ^{\tau \omega } \hat{z}_\lambda ^{\tau \omega } \ge \underset{z}{\text {minimize}}&{\sum _{\{\lambda \in \Lambda \, :\, o_\lambda = o\}}\ell ^{\tau \omega }_{\lambda } z^{\tau \omega }_{ \lambda } } \\ \text {subject to}&{\sum _{\{\lambda \in \Lambda \, :\, o_{\lambda } = o\}}z^{\tau \omega }_{ \lambda }}{= 1,} \\&{\sum _{\{\lambda \in \Lambda \, :\, o_\lambda = o, \, a \in \lambda \}}\hspace{-0.7cm}z^{\tau \omega }_{ \lambda }}{\le \hat{y}^\tau _a, \quad \forall \ a \in \mathcal {A},} \\&{\sum _{\{\lambda \in \Lambda \, :\, o_\lambda = o, \, f_\lambda = f\}}\hspace{-0.9cm}z^{\tau \omega }_{ \lambda } \!\le \hat{x}^{\tau }_{f}, \quad \forall \ f\!\in \mathcal {F},} \\&{z^{\tau \omega }_{ \lambda }}{\ge 0 \quad \forall \ \lambda \in \Lambda \, :\, o_\lambda = o.} \end{aligned}$$

By duality, we obtain the following equivalent inequality:

$$\begin{aligned} \sum _{\{\lambda \in \Lambda \, :\, o_\lambda = o\}} \ell _\lambda ^{\tau \omega } \hat{z}_\lambda ^{\tau \omega } \ge \underset{\alpha ,\beta ,\gamma }{\text {minimize}}&{\alpha _o^{\tau \omega } + \sum _{a \in \mathcal {A}} \beta _{oa}^{\tau \omega }\hat{y}_a^{\tau } + \sum _{f \in \mathcal {F}} \gamma _{of}^{\tau \omega } \hat{x}_f^\tau } \\ \text {subject to}~&{\alpha _o^{\tau \omega } \!+\! \sum _{a \!\in \! \lambda }\beta _{oa}^{\tau \omega } \!+\! \gamma _{of_\lambda }^{\tau \omega } \!\le \! \ell ^{\tau \omega }_{\lambda },}{\ \forall \ \lambda \!\in \! \Lambda \ :\ o_\lambda \!=\! o,} \\&{\beta _{oa}^{\tau \omega } \le 0,}{ \ \forall \ a \in \mathcal {A},} \\&{\gamma _{of}^{\tau \omega } \le 0,}{ \ \forall \ f \in \mathcal {F}.} \end{aligned}$$

Thus, we obtain that for any $(\alpha ,\beta ,\gamma )$ satisfying Eqs. (9-11), the following inequality holds:

$$\begin{aligned} \sum _{\{\lambda \in \Lambda \, :\, o_\lambda = o\}} \ell _\lambda ^{\tau \omega } \hat{z}_\lambda ^{\tau \omega } \ge \alpha _o^{\tau \omega } + \sum _{a \in \mathcal {A}} \beta _{oa}^{\tau \omega }\hat{y}_a^{\tau } + \sum _{f \in \mathcal {F}} \gamma _{of}^{\tau \omega } \hat{x}_f^\tau . \end{aligned}$$

Since constraints Eqs. (9-11) do not depend on $(\hat{u},\hat{v}, \hat{x}, \hat{y}, \hat{z})$, we deduce that Eq. (8) is a valid inequality for the MIP Eq. (1). □

From Proposition 2, we can generate these decomposition-based valid inequalities (or equivalently cuts) to speed up the column generation algorithm when processing tree nodes. Specifically, within the B&P algorithm (Algorithm 2), we introduce a global cutset $\mathcal {C}$ that is added to the master problem $\mathcal {L}(\mathcal {B},\Lambda )$ at each tree node. Every time we process a node and the column generation algorithm terminates with an integer-feasible solution, we extract the optimal dual variables $\hat{\alpha }_o^{\tau \omega }$, $\hat{\beta }_{oa}^{\tau \omega }$, and $\hat{\gamma }_{of}^{\tau \omega }$ respectively associated with Eqs. (1i), (1j), and (1k), for every $o\in \mathcal {O}$, $f \in \mathcal {F}$, $a \in \mathcal {A}$, $\omega \in {\Omega }$, and $\tau \in \mathcal {T}$. Such optimal dual variables naturally satisfy Eqs. (9-11) and we add the corresponding decomposition-based cuts Eq. (8) to the cutset $\mathcal {C}$, which will be applied to all subsequent tree nodes.

We note that adding the cutset $\mathcal {C}$ to each master problem impacts reduced cost computations during column generation. Nonetheless, we still find that the pricing problems can be solved using shortest-path algorithms. In Appendix B.2, we discuss refinements to the process of generating cuts. First, we consider that cuts of the form Eq. (8) may be dominated by previously generated cuts. Therefore, we provide an algorithm to generate a set of non-dominated cuts to be added to the master problem at each tree node. Second, adding cuts early in the algorithm can further accelerate its convergence. Thus, we aim to derive cuts from the integer feasible solution identified via the heuristic from §3.4.1. However, dual variables cannot be computed using complementary slackness and are not directly available. In Appendix B.2, we show how to analytically derive cuts from only first-stage variables.

## Computational study

In this section, we analyze the computational performance of our proposed algorithm on synthetic test cases. The primary purpose of these experiments is to evaluate the efficacy of our approach as a function of network size and to quantify the benefit of the valid inequalities derived in §3.4.3. Our secondary purpose is to identify the parameters of Eq. () to which the performance of the proposed approach is sensitive. Lastly, we compare the performance of our exact method to that of a variable neighbor search (VNS), which heuristically solves an edge-based formulation.

### Test instances

We generate a portfolio of random test instances based on methods used in prior literature [22, 23, 25]. First we fix the number of nodes, $|\mathcal {N}|$, number of candidate facilities, $|\mathcal {F}|$, and number of time periods, T. Then, we randomly distribute $|\mathcal {N}|$ nodes across a square grid and sample $|\mathcal {F}|$ of these nodes to be the set of candidate facilities. We assume that $\mathcal {O}= \mathcal {N}$, namely, patients can originate from any node. To generate the road network, we first construct a random adjacency matrix such that nodes that are closer to each other are more likely to have an arc connecting them than nodes that are farther away from each other. We then randomly assign the surface type for each road from a set of $|\mathcal {S}|=5$ surface types, with lower-quality types being more likely than higher-quality types. In so doing, we construct the road network available at the beginning of the planning horizon. Note that the graph $\mathcal {G}= (\mathcal {N},\mathcal {A})$ of potential improved road networks can be obtained by making a copy of each road with different surface types, thus generating the set of type-dependent arcs $\mathcal {A}$. We also generate $|\Omega |= 2^{T}$ event scenarios corresponding to a rainy and dry event for each time period. For brevity, we defer additional details regarding the random generation of the adjacency matrix, surface types, and other model parameters to Appendix C.

In total, we construct a set of 360 test instances. These 360 test instances result from varying the the number of nodes in the network to 4 different values ($|\mathcal {N}| \in \left\{ 10,20,50,75\right\}$) and the number of candidate facilities to 3 different values ($|\mathcal {F}| \in \left\{ \lfloor \frac{1}{4}\cdot |\mathcal {N}|\rfloor ,\lfloor \frac{1}{2} \cdot |\mathcal {N}|\rfloor ,\lfloor \frac{3}{4} \cdot |\mathcal {N}|\rfloor \right\}$). Then, for each of the 12 ($|\mathcal {N}|,|\mathcal {F}|$) combinations, we generate 30 different instances according to the procedure above. We fix T=5 for all instances.

**Fig. 2:**
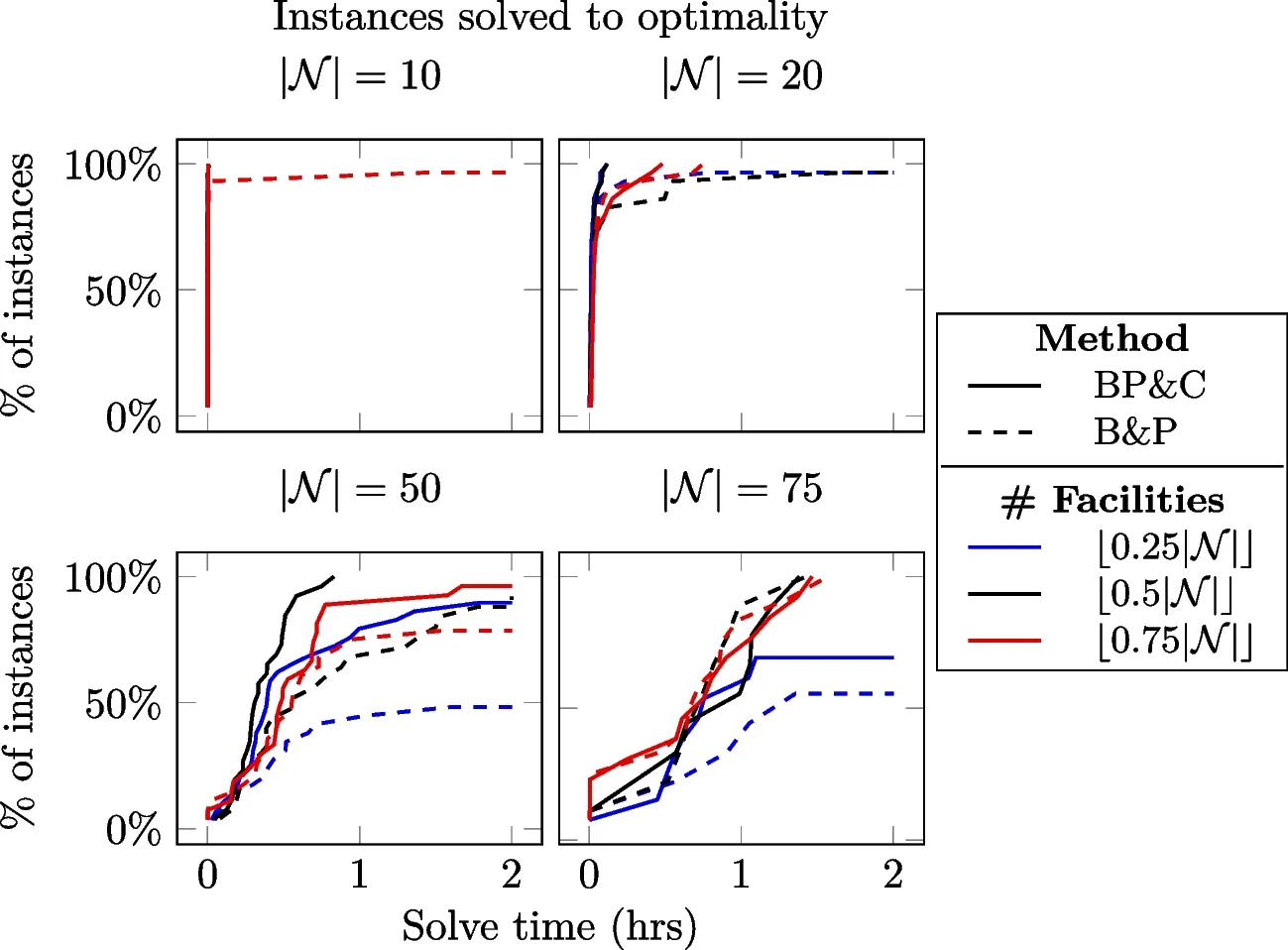
The cumulative percentage of instances solved to optimality within the given time for our BP&C algorithm (solid lines) compared to the B&P approach described in §3 without the cuts described in §3.4.3 (dashed line). Line color is based on the fraction of nodes that have facilities. The maximum allocated compute time was 120 minutes. The results are faceted by the number of nodes in the instances, $|\mathcal {N}|$. Each line is composed of the outcomes of 30 separate instances with the same ($|\mathcal {N}|, |\mathcal {F}|$) parameters. The same instances are tested across solution methods

For each instance, we calculate the total cost of expanding all candidate facilities during the first time period, $\bar{C}:=\sum _{f\in \mathcal {F}} c_{f}^{1}$. Then, we assume that a certain proportion, κ, of $\bar{C}$ would be divided equally across all time periods so that $C^{\tau } = \kappa \cdot \frac{\bar{C}}{T}$ for all $\tau \in \mathcal {T}$. Similarly, we calculate $\bar{H}:=\sum _{f\in \mathcal {F}} h_{f}^{1}$ and set $H^{\tau } = \kappa \cdot \frac{\bar{H}}{T}$ for all $\tau \in \mathcal {T}$.

We conduct three sets of experiments. In the first set of experiments, we explore the impact of problem size on solution times, considering all 360 random instances for a fixed value of $\kappa =50\%$. In the second set of experiments, we only consider 15 random instances for which $|\mathcal {N}| = 50$ and vary the budgets by considering $\kappa \in \{10\%,20\%,\ldots ,100\%\}$. We hypothesize κ may impact the performance of our solution method.

In the first two experiments, we determine the benefit of adding the cuts presented in §3.4.3 to our algorithm. That is, we compare our complete BP&C to the approach outlined in §3 but which omits the cuts described in §3.4.3. We refer to the former approach as BP&C and hereafter refer to the later as B&P. We assume that both algorithms employ the heuristically generated warm start from §3.4.1 and the special ordered set branching rule from §3.4.2.

In the third set of experiments, we compare our exact solution methodology to the performance of a widely used heuristic for the general facility location network design problem: VNS. VNS was first introduced for the general facility location network design problem by Cocking [37], but has since been further developed by Ghaderi [22]. These VNSs typically first solve the facility location problem assuming a fixed network, and then solve the network design problem with those facilities fixed. Then, the heuristic treats the facility location solution as a vector in a normed linear space and sequentially searches among “similar" solutions (defined as being within a distance *R* of the current solution) that improve upon the current solution. We compared our solution method to a VNS designed for a general multi-period FLNDP [22]. This VNS was developed using an edge-based formulation, heuristically fixing the same first-stage variables as in () and solving an optimization model for each community to determine patient flows along each edge. We provide a full edge-based formulation in Appendix D: (D1a).

The VNS of Ghaderi [22] was designed for problems with separate budgets for the road improvements and facility improvements, and they do not consider stochastic events. Therefore, we modified their algorithm to our context. Specifically, we proportioned the total budget $C^{\tau }$ into two separate budgets: a road improvement budget, $C_{\mathcal {A}}^{\tau }$, and a facility improvement budget, $C_{\mathcal {F}}^{\tau }$. We consider that the ratio of the total budget that can be spent on road improvements, ${C_{\mathcal {A}}^{\tau }}/{(C_{\mathcal {A}}^{\tau }+C_{\mathcal {F}}^{\tau })}$, is an input parameter to the heuristic. To handle stochastic scenarios we introduce a set of ω specific second-stage decision variables and minimize in expectation, weighting each $\omega \in \Omega$ by $p^{ \omega }$. A complete description of this modified VNS heuristic is available in Appendix D.

All algorithms are implemented in Python 3.11. We solve all optimization problems using Gurobi v.11.0.2, running at 3.0 Ghz (1 core) with 32 GB of assigned RAM and Red Hat Linux v.8.10. We specify a time limit of 120 minutes for all instances. When solving for the true optimal solution in the third set of experiments, we did not specify a time limit.

### Results

Figure 2 presents the results from our first set of experiments. When the number of nodes in the network is relatively small (i.e., $|\mathcal {N}|\in \{10,20\}$), we observe that the BP&C algorithm is able to solve all instances to optimality within 28 minutes. Meanwhile, the B&P algorithm is able to solve most, but not all, of the instances within the time limit. For the larger network sizes that we tested (i.e., $|\mathcal {N}|\in \{50,75\}$), we observe that the BP&C algorithm solves at least as many instances as the B&C algorithm within the time limit for all values of $|\mathcal {F}|$. This trend is evident in Fig. 2 as the solid lines (representing BP&C) frequently appear above the dashed lines (representing B&P), indicating a higher fraction of instances solved. For example, with 50 nodes, BP&C solves 26 (87%) of the 30 instances with the fewest number of facilities, but B&P only solves 14 (47%) of the same instances (see the blue lines in Fig. 2 for $|\mathcal {N}|=50$). These findings demonstrate that there is value to adding the cuts into our B&P algorithm.

**Fig. 3:**
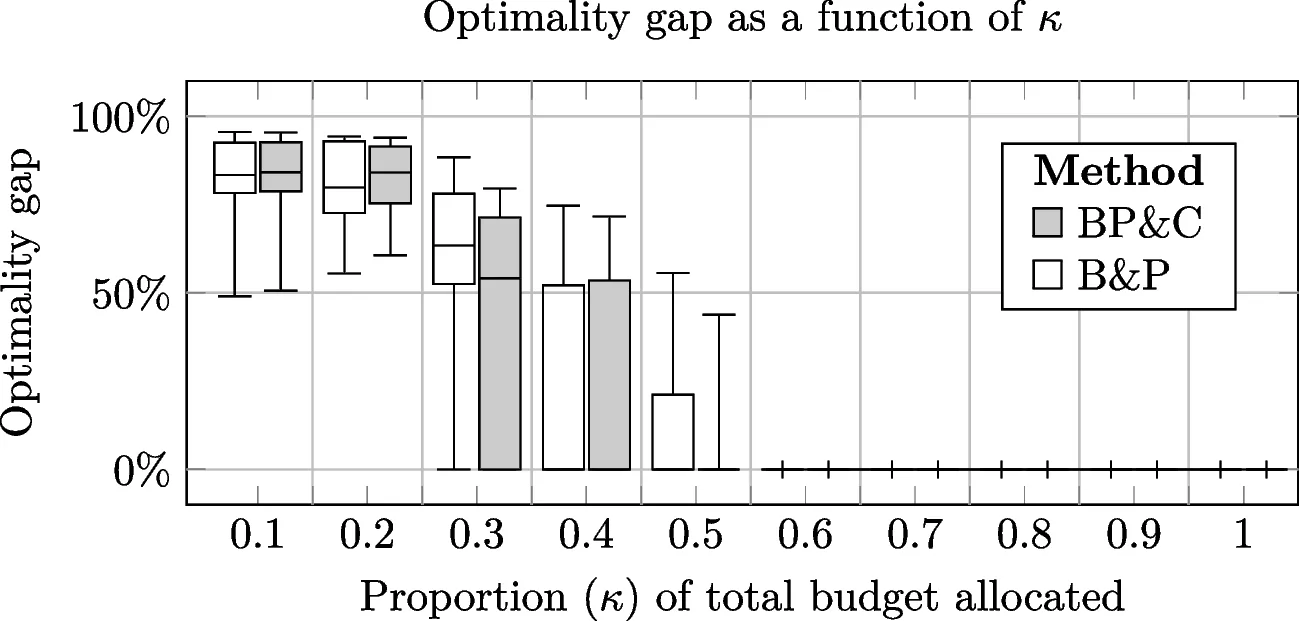
Among 15 instances with $|\mathcal {N}| = 50$ we vary κ, the fraction of the total construction and staffing costs allotted for C and H, from 10% to 100%. Each bar consists of 15 instances which are each allocated 120 minutes of solve time

Figure 3 illustrates the results of our second set of experiments that examine the sensitivity of the performance of our algorithms to the values of our budget parameters, C and H. For the smallest tested values of $\kappa =10\%$, which correspond to the smallest tested monetary and staffing budgets for each instance, both the BP&C and the B&P struggle to obtain an optimality gap under 50%. When we consider κ in the range of 30%-50%, there are noticeable differences in performance between BP&C and B&P. For example, when $\kappa = 30\%$, the BP&C algorithm solves 4 of the 15 instances to optimality, but the B&P algorithm can only solve one instance to optimality within the time limit. When $\kappa \ge 60\%$, both methods are able to solve all instances to optimality. These results suggest that solving versions of the facility location network design problem (FLNDP) with tight budgets is challenging, as they require a more complex budget allocation to achieve optimality. On the other hand, both solution methods perform well when budgets are less restrictive. Importantly, the BP&C algorithm outperforms the B&P algorithm for moderate budget levels.

Table 3 presents the results of the third set of experiments, comparing our BP&C approach with the VNS heuristic adapted from the literature (see Appendix D). We find that VNS provides high-quality solutions quickly, but is sensitive to the choice of $C_{\mathcal {A}}^{\tau }$. On small instances, both BP&C and VNS are able to solve all instances to near optimality quickly. Further, as the problem size grows, the VNS solution quality rapidly deteriorates as shown by increasing gaps to the optimal solution. VNS is sensitive to the relative ratio of $C_{\mathcal {A}}$ and $C_{\mathcal {F}}$, and fine-tuning the heuristics performance would require the user to optimize this meta-parameter. This is a notable difference from the BP&C approach, which does not require any meta-parameters. Lastly, VNS is unable to provide an optimality gap at termination, whereas BP&C is able to provide one during execution.

In summary, we find that BP&C outperforms B&P and that the difference in performance is most pronounced in our test cases with larger network sizes and moderate budgets. We also observe that instances where $|\mathcal {F}| \ll |\mathcal {N}|$ or $\kappa < 30\%$ are the most challenging for both solution methods. Conversely, the instances with the smallest networks and those with the highest values of κ are quickly solved regardless of the number of facilities considered. We also find that an adaptation of an existing VNS heuristic provides good solutions to small instances quickly. When compared to our BP&C approach, the VNS generally provides worse solutions, but delivers these solutions faster.

## Case study: geographic access to cancer care in Rwanda

In this case study, we explore how both expansions of facilities and improvements to the road network might improve access to cancer care in Rwanda. We begin by providing some context about the current state of cancer care in Rwanda, a low-income country in East Africa, which in many ways exemplifies the challenges in geographic access to cancer care. Then, we will describe the data used to parameterize our model, the policies we compared, and the results of using our model to examine how cancer care in Rwanda might be improved under different monetary and human capital budgets.

**Table 3:** Comparison of the VNS approach and the BP&C algorithm, with solve time limited to 120 minutes. True optimal solutions were obtained via BP&C without a limit on compute time. Optimality gaps (Opt. Gap) and solve times are reported as median, minimum and maximum values

$\mathcal {N}$	${C_{f}^{\tau }}/{C^{\tau }}$	Variable Neighbor Search						Branch Price and Cut					
		Opt. Gap (%)			Solve time (s)			Opt. Gap (%)			Solve time (s)		
		med.	min	max	med.	min	max	med.	min	max	med.	min	max
10	1/4	14.8	0.3	33.0	0.3	0.1	2.0	0.0	0.0	0.0	4.4	0.1	24.8
	1/2	17.6	0.0	38.0	0.1	0.1	0.6						
	3/4	15.7	0.0	34.1	0.1	0.0	0.3						
	1	12.8	0.0	33.8	0.1	0.1	0.5						
20	1/4	15.8	5.4	39.1	0.5	0.2	7200	8.5	0.0	71.6	951.7	5.8	7200
	1/2	32.0	3.3	49.7	0.3	0.1	69.9						
	3/4	35.0	1.9	66.7	0.3	0.1	4.4						
	1	34.0	0.1	66.6	0.3	0.1	2.4						
50	1/4	11.1	6.6	13.1	6.8	0.6	21.9	10.6	0.0	50.0	3397.0	456.5	7200
	1/2	15.0	12.1	38.3	5.7	0.4	7200						
	3/4	42.2	14.6	55.9	1.9	0.6	7200						
	1	56.7	12.2	71.9	1.0	0.4	1170.7						
75	1/4	14.3	13.4	21.6	71.3	1.0	1784.3	10.9	0.0	36.8	4884.7	1436.8	7200
	1/2	24.8	22.9	30.5	10.2	0.8	3328.4						
	3/4	35.3	30.8	44.4	1.8	0.8	7200						
	1	62.4	29.9	82.6	1.1	0.5	5889.2						

In 2022, it was estimated that more than 7,000 people died from cancer in Rwanda, a country that only has 13.6 million people [2]. Although cancer data is limited, these data indicate that Rwanda primarily treats late-stage cancers [11] and that patients located furthest from care are more than two times as likely to have a late-stage diagnosis compared to their peers who live close to care [38]. At the time of writing, Rwanda does not have a comprehensive oncologic registry, making it difficult to understand spatial demand for cancer services.

**Fig. 4:**
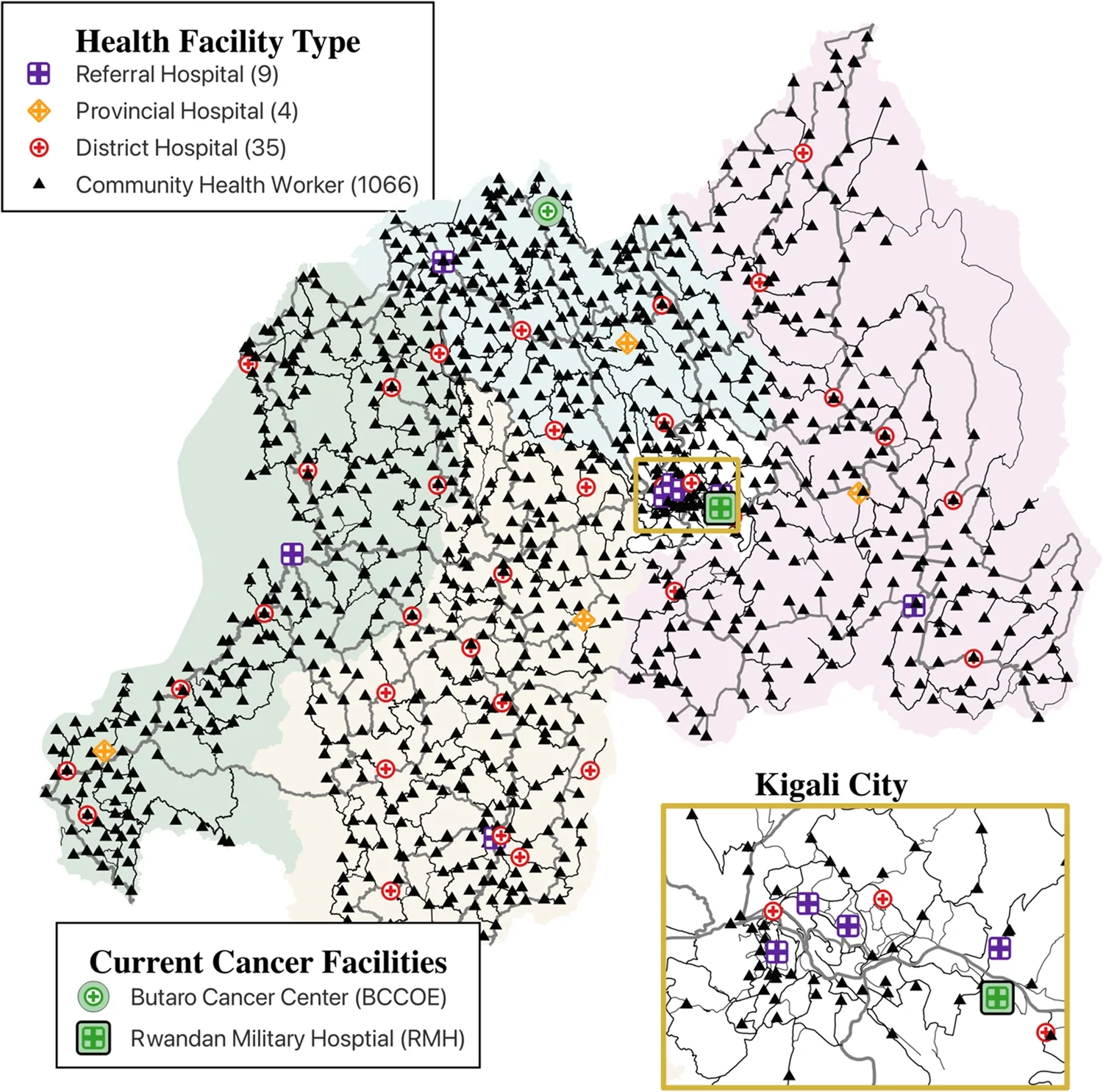
Rwandan instance with community health workers posts and hospital locations shown, background colored by province, with facility number displayed in legend. Both publicly accessible cancer hospitals, the Rwandan Military Hospital and Butaro Cancer Center of Excellence, are shown in green. Candidate facilities ($\mathcal {F}$) are shown as colored with crosses. Roads are illustrated as black lines

Rwanda’s tertiary cancer care services are not distributed uniformly throughout the country. While basic pathology and radiology diagnostics are available at all district and provincial hospitals in Rwanda, there are only three hospitals that offer tertiary cancer care services. Two of these hospitals are public hospitals—the Rwandan Military Hospital (RMH) and the Butaro Cancer Center of Excellence (BCCOE)—and the third is a private hospital—the King Faisal Hospital. RMH is located in Rwanda’s capital, a city of approximately 1.8 million people that is located in the middle of the country. Cancer services at RMH are provided by the Rwandan Government. BCCOE is located in the far north of the country’s Northern Province, approximately 120 kilometers by road from Kigali. BCCOE is owned and operated by Partners in Health, an American non-governmental organization whose mission is to deliver health care “to the world’s poorest places.” The location of BCCOE was meant to provide excellent cancer care to a primarily rural population. We focus on access to public hospitals offering tertiary cancer care. All Rwandans participate in a collective insurance scheme. While some work by Rubagumya et al. [39] has detailed that the cost of care likely presents a burden, the collective insurance scheme makes care at public hospitals more financially accessible than private ones. The geographic location of the public hospitals offering tertiary cancer care is shown in Fig. 4.

Traveling to these tertiary cancer care services can be challenging. Rwanda is known as “the land of a thousand hills” due to its hilly and mountainous terrain, especially in its central and western regions. Moreover, Rwanda has two three-month rainy seasons, during which the rain can be heavy and persistent. Some regions in Rwanda receive over 1500 mm (approximately, 59 inches) of annual rainfall concentrated in a few months [40]. Flooding and landslides during the rainy seasons are common, can be fatal, and frequently damage infrastructure [41–43]. Together, this makes travel along unpaved roads quite challenging, and certain parts of the road network can require long travel times to traverse.

Because of the locations of the existing tertiary cancer care services, there is significant variation in how long it takes Rwandans to access this type of cancer care. Less than 5% of the population within the capital province have to travel more than two hours to reach a comprehensive cancer center. Meanwhile, more than 95% of the population in the rural Western Province has to travel more than two hours to access this type of care [7]. These disparities in access are associated with disparities in the receipt of cancer care: rural regions in Rwanda have later diagnoses and experience longer diagnostic and treatment delays [11].

Previous studies have explored potential plans to expand tertiary cancer care to reference hospitals in Rwanda. [7] evaluated a policy that expanded tertiary cancer care at all reference hospitals and showed that this plan would increase the percentage of rural Rwandans living within one hour of care from 8% to 33%. Sapirstein et al. [8] used an optimization approach and demonstrated that other selections of reference and provincial hospitals to expand care could result in better access at lower cost. However, even with the highest levels of investment in cancer care, they demonstrate that around 20% of Rwandans would still live more than two hours from tertiary cancer care. In our case study, we examine the extent to which simultaneously expanding healthcare facilities and improving the road network could improve access to cancer care in Rwanda, given these barriers to geographic access.

**Table 4:** Summary of the estimates of model parameters and their corresponding data sources for the case study

Set/Parameter	Description of estimate(s)	Source
$\mathcal {N}$	All population centers, road junctions, and health facilities in Rwanda	[15, 44]
$\mathcal {F}$	Existing district, provincial, and reference hospitals	[7, 44]
$\mathcal {O}$	The spatial distribution of Rwanda’s population aggregated to the closest community health worker	[8, 44]
$\mathcal {S}$	The five road surfaces as defined in Table 5	[15]
$\mathcal {A}$	Rwanda’s road network extracted from OSM	[15]
$\mathcal {T}$	$\left\{ 1,2,3,4,5\right\}$; a five-year planning period corresponding to the MOH’s cancer planning periods	[12]
Ω	$\{$dry, rainy$\}{^{T}}$; the dry & rainy season in each time period τ	Assumption
$c^\tau _{a}$	The cost to improve a road depends on its current surface type; estimated from the Rwandan Transport Ministry	[45]
$$c^\tau _f$$	$12.9 million if f is currently a reference or provincial hospital; $26.5 million if f is a district hospital	[46]
$h^\tau _f$	3 oncologist teams if f is either a reference and provincial hospital; 6 oncologist teams if f is a district hospital	[8]
$$C^{\tau }$$	Varies from $0 to $25 million in test cases	
$H^{\tau }$	Varies from 2 to 6 in test cases	
$p^{ \omega }$	Each year has independent 30% probability of rainy event: $\prod _{\tau \in \mathcal {T}} (0.7 \cdot 1\!\!1_{\omega _{\tau } = \text {dry}} + 0.3 \cdot 1\!\!1_{\omega _{\tau } = \text {rainy}})$	[40]
$d_o^{\tau \omega }$	Proportional to the number of people in population center o, for all τ and ω	[2, 8, 47]
$\ell ^{\tau \omega }_{a}$	Based on the estimated speeds outlined in Table 5	[7, 45, 48, 49]

### Model parameterization

We construct an instance of () that could be used to help the Rwandan Government and Ministry of Health (MOH) plan its expansion of tertiary cancer care over a five-year planning horizon to be consistent with its five-year cancer control plans [12]. A summary of our data sources is provided in Table 4. Our constructed instance is shown visually in Fig. 4.

First, we construct the set of population centers in our model and their associated demand for cancer care services. We use the methodology described in Sapirstein et al. [8] to estimate population density from satellite data. This process results in a continuous spatial distribution of the population across Rwanda. To create a representative discrete set of population centers, $\mathcal {O}$, we assign population density to its closest community health worker post in Rwanda. Using AccessMod5 [47], we estimate that 50% Rwandans are located within 11 minutes of their nearest CHW, with 75% located within 19 minutes and 95% located within 34 minutes. We assume that the prevalence of cancer is uniformly distributed across the population of Rwanda. Thus, the total demand for cancer care from a given population center o is proportional to its population. We also assume that the demand for cancer care, $d^{\tau \omega }_{o}$, is constant across all time periods $\tau \in \mathcal {T}$ and weather scenarios $\omega \in \Omega$ (which we define later).

We consider that the Rwandan MOH may wish to expand tertiary cancer care to hospitals that currently offer one of three levels of care (district, reference, and provincial). These existing hospitals form our candidate set of facilities, $$\mathcal {F}$$. We obtain the locations of these candidate facilities from Rwanda’s MOH through Rwanda’s Africageoportal website [44]. Consistent with prior literature, we estimate that the cost to expand tertiary cancer care at a reference or provincial hospital is $12.9 million for reference and that the cost to expand this care to a district hospital is $26.5 million USD [8, 46]. These costs represent initial costs associated with adding cancer services. Ongoing costs of providing cancer care were excluded due to a lack of data availability. We estimate that adding services to reference and provincial hospitals would require 3 additional oncologist teams and that district hospitals would require 6 such teams. That is, $$h^{1}_{f} = 3 $$ if f is either a reference or provincial hospital and $$h^{1}_{f} = 6$$ otherwise.

We also consider that the Rwandan government may wish to improve its road network to improve access to care. We obtain data to construct a representation of the road network from OpenStreetMaps (OSM) as of March 2025. The existing surfaces of these roads are labeled to be consistent with OSM’s classification. This process results in 5 road surface types, which are described in Table 5. To estimate the cost to improve a road between *i* and *j* from surface *s* to s+1, we consider that there is a per-kilometer cost to upgrade and that the total cost is proportional to the total distance between *i* and *j*. These surface-specific per-kilometer road construction costs are estimated from the Rwandan Ministry of Transport [45].

**Table 5:** The types of road surfaces considered in our case study extracted from OpenStreetMaps (OSM) [15] with their corresponding speeds during the dry and rainy seasons [7, 48, 49]

$${s}$$	Road Surface	OSM Classification	Speed (km/hr)		Cost$${}^{*}$$
	Description		Dry seasons	Rainy seasons	($1,000/ km)
1	All other roads	Unclassified	10	6	-
2	Residential streets	Tertiary & residential	18	11	97
3	Minor highway	Secondary	27	16	97
4	Highways	Primary	40	37	151
5	Major highways	Trunk & motorway	60	54	793

${}^*$Costs are estimated from [45] and converted into United States dollars from Rwandan Francs

We consider that, each year, we have two distinct weather scenarios: the rainy seasons and the dry seasons. Across each year, there is a 30% chance of being in the rainy season and a 70% chance of being in the dry season. We construct Ω by considering all possible rainy/dry combinations of length T. The time to travel along each road surface depends on the season. During the rainy seasons, the speed that a patient can travel along a road is significantly slower than the speed achievable during the dry season. The gap between the speed during the rainy season and dry season is more pronounced for lower-quality roads, such as gravel and dirt roads, due to mud and washout (see Table 5, [48, 49])

Using the instance described above, we consider several different test cases that vary in terms of the monetary budget $$C^{\tau }$$ and the staffing budget $$H^{\tau }$$ for each period τ. We vary the annual monetary budget for Year 1 from $0 to $25 million in $5 million dollar increments. Independently, we also consider three different annual staffing budgets, $$H^\tau $$: 2, 4, and 6 new oncologist teams for each $$\tau \in \mathcal {T}$$. The monetary cost estimates and budgets described above for Year 1 are adjusted using a 5% annual inflation rate to obtain the corresponding cost estimates in future time periods (i.e., $$\tau =2,\ldots ,5$$).

**Table 6:** The average travel time required to access tertiary cancer care under the FLNDP policy and the FLP policy in each of our test cases, across a five-year planning horizon

Test	Budget		Travel Time by Season (minutes)					
Case	Monetary	Human	FLNDP			FLP		
	(mil. USD)	Capital	Dry	Rainy	Average	Dry	Rainy	Average
0${}^{*}$	-	-	162.8	407.0	210.7	162.8	407.0	210.7
1	5	2	104.6	261.5	151.7	116.0	290.1	168.2
2		4	104.6	261.5	151.7	116.0	290.1	168.2
3		6	104.6	261.5	151.7	116.0	290.1	168.2
4	10	2	101.7	254.3	147.5	116.0	290.1	168.2
5		4	81.3	203.1	117.8	90.5	226.3	131.3
6		6	81.3	203.2	117.8	89.9	226.3	130.4
7	15	2	100.8	252.0	146.2	116.0	290.1	168.2
8		4	79.8	199.4	115.7	90.7	226.8	131.5
9		6	69.1	172.8	100.2	76.6	191.6	111.1
10	20	2	100.1	250.2	145.1	116.0	290.1	168.2
11		4	79.2	197.9	114.8	90.8	227.1	131.7
12		6	66.1	165.1	95.8	73.9	184.8	107.2
13	25	2	99.5	248.7	144.2	116.0	290.1	168.2
14		4	78.3	195.6	113.5	90.5	226.3	131.3
15		6	66.1	165.3	95.9	75.3	188.3	109.2

$^{*}$ Test case 0 represents the current state of access; FLNDPFLP. Each test case varies in terms of monetary and staffing budget. The travel times are reported as a five-year average for the rainy seasons, the dry seasons, and overall average (in minutes)

### Policy comparisons and outcome measures

Using the instances of () described above, we compare two policy approaches to expand access to cancer care in Rwanda. The first approach is to consider only improvements to healthcare infrastructure. To optimize planning using this approach, we solve (), the facility location problem (FLP) in which only facility expansions are considered; no improvements to the road network are considered. The second approach is to consider improvements to both the healthcare infrastructure and the transportation infrastructure using our proposed FLNDP model. We solve this large-scale FLNDP using our BP&C algorithm described in §3. For brevity, hereafter we refer to these two approaches as the “FLP policy” and the “FLNDP policy,” respectively.

To compare these two policies, we calculate the travel time required to access tertiary cancer care on average and by quartile of current travel time, in both the rainy and the dry seasons. We also visualize the changes in travel time to better understand which populations see the benefits of improved access under these different policies. Further, we investigate when an FLNDP policy offers the most benefit relative to an FLP policy. To quantify these benefits, we describe the reduction in travel times that is directly attributable to road network improvements. To do so, we compare the travel times to the facilities expanded under the FLNDP policy in the current road network to the travel times to the facilities expanded under the FLNDP policy in the FLNDP policy’s improved road network. This comparison enables us to determine how much of the reduction in travel time is attributable to facility expansions versus road network improvements.

### Results

Using the instances of Eq. () described above, we evaluate the performance of the two policies for expanding access to cancer care in Rwanda. The processes described above result in instances consisting of a network with $|\mathcal {N}|= 3{,}597$ nodes, $|\mathcal {F}|= 48$ candidate facilities, $|\mathcal {O}|= 1{,}066$ population centers, $|\mathcal {S}|=5$ surface types, $|\mathcal {A}|= 7{,}644$ type-dependent arcs, T=5 time periods, and |Ω| = 32 stochastic scnearios. We are able to solve the FLP to optimality across all test cases, and We are able to solve the FLP (Eq. 6) to optimality across all test cases, and FLNDP (Eq. 1) to within $4.6\%-14.7\%$ of optimality for all test cases.

**Fig. 5:**
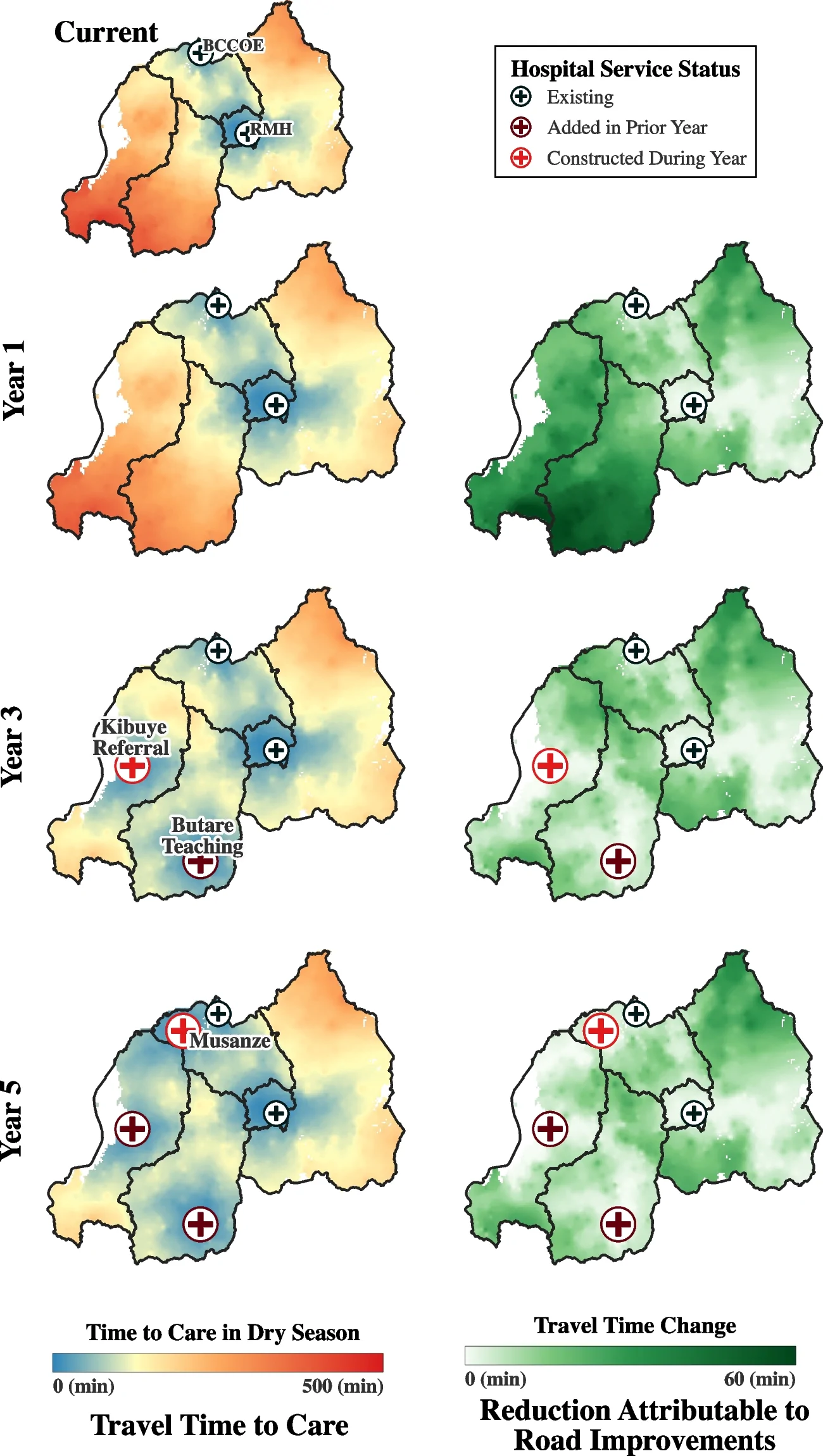
An illustration of how the optimal expansion of facilities and road network improvements change travel times to access tertiary cancer care in Rwanda over the five-year planning horizon. Facilities are indicated with the + symbol. Existing facilities are colored black, facilities expanded during the corresponding year are colored red, and facilities expanding during a previous year are colored Maroon. (Left) The travel time required to access care by population center by year. (Right) The reduction in travel time that is directly attributable to road network improvements, by year

Table 6 presents the average travel time required to access tertiary cancer care under the FLNDP policy and the FLP policy in each of our test cases. These travel times are reported for the rainy seasons, dry seasons, and on average. With the current facilities and road network, the travel time to tertiary cancer care is approximately 3.5 hours on average. However, the current travel time to care is more than 6.5 hours in the rainy seasons. For each tested combination of monetary and staffing budget, the FLNDP policy results in lower average travel times, driven in large part by reductions in the travel times during the rainy seasons. For example, when the annual monetary budget is $5 million and in all staffing budget levels, the FLNDP policy achieves an average travel time of 151.7 minutes compared to the FLP policy’s average travel time of 168.2 minutes. This difference is due to a 11.2-minute difference during the dry seasons (104.6 minutes under the FLNDP policy compared to 116.0 minutes under the FLP policy) and a 28.6-minute difference in the rainy seasons (261.5 minutes under the FLNDP policy compared to 290.1 under the FLP policy). These trends persist as the monetary and staffing budgets increase, but are most pronounced for high monetary budgets and low staffing budgets. For example, for our highest monetary budget considered ($25 million) and the lowest staffing budget (two oncologist teams per year), the FLNDP policy achieves an average travel time that is 24 minutes shorter than the FLP policy, and this improvement is in large part because the FLNDP policy has an average travel time that is 41.4 minutes shorter than the FLP policy during the rainy seasons.

**Fig. 6:**
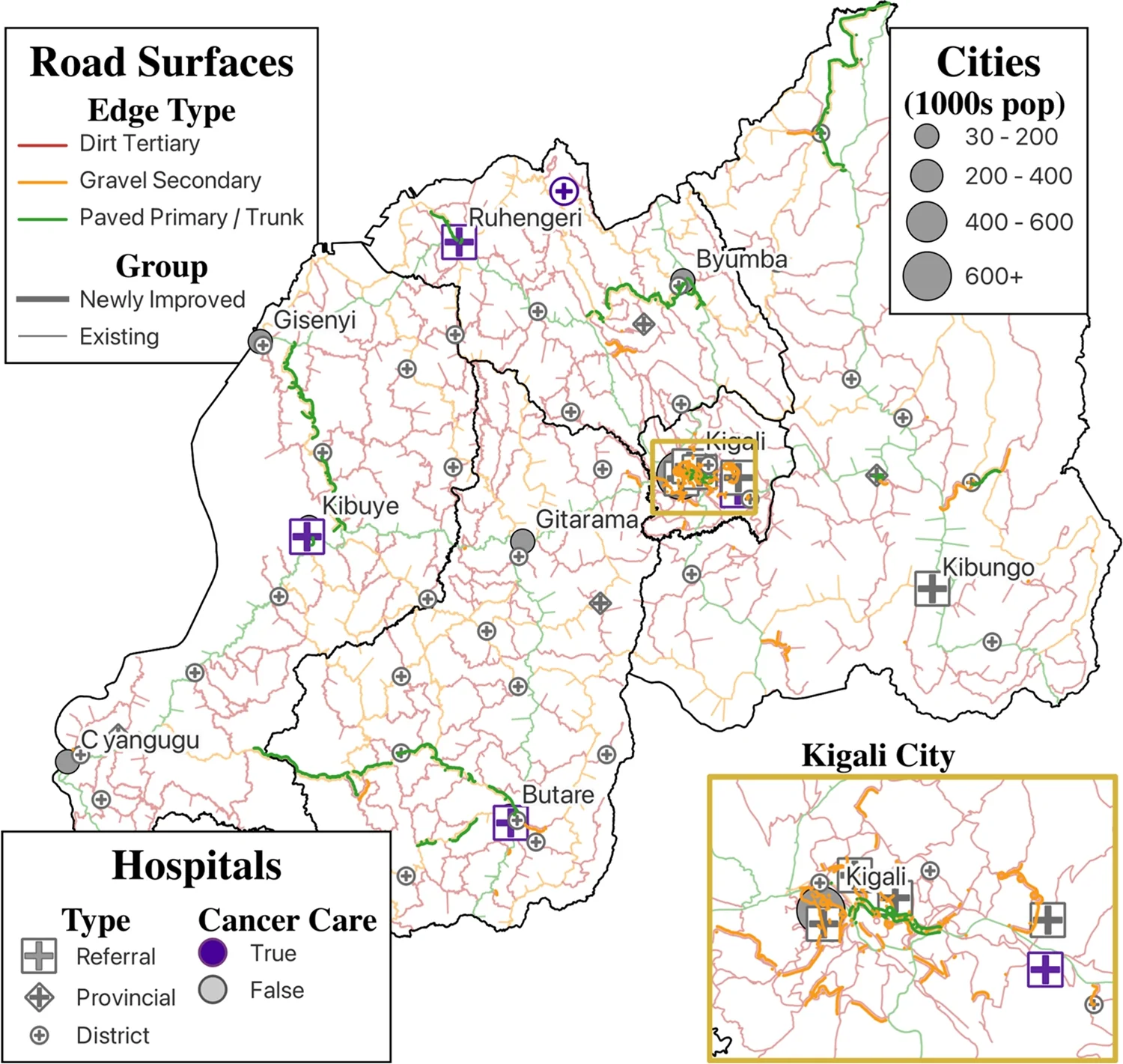
Illustration of improved arcs by end of 5-year decision horizon, under $5 million and 4 additional oncologist per year, as shown in Fig. 5. Bold arcs indicate improvements, with color indicating final surface type. Large Rwandan cities are labeled on map, with locations and populations from Esri environment systems research institute. Additionally, we show facilities, with shape corresponding to the type of hospital. Purple hospitals provide cancer care by the end of the decision horizon. Gray indicates a hospital without cancer care at the end of the decision horizon

We now present detailed results for a representative test case: test case 2. We select this test case to explore in detail because the monetary budget of $5 million USD closely resembles historical trends in cancer care spending [46] and 4 new oncologist teams per year is an optimistic estimate of the impact that new training programs may have [50]. Figure 5 illustrates the optimal facility expansions over the planning horizon in this representative test case. In this test case, the FLNDP and FLP policies recommend the same facility expansions during the same time periods (as illustrated in the top of Fig. 5). In Year 2, both policies recommend expansion of the University Teaching Hospital of Butare (located in the Southern Province) and, in Year 3, they both recommend expansion of the Kibuye Referral Hospital (located in the Western Province). Due to budget constraints, neither policy is able to recommend opening hospitals during Year 4. In Year 5, both policies recommend that the Musanze Referral Hospital (located in the Northern Province) be expanded. Under the FLNDP policy, many graveled minor highways in the Southern Province are improved to be paved highways during Year 1. Later, we observe that the FLNDP policy recommends more roads in the Eastern Province to be improved. Interestingly, we also observe that the FLNDP policy recommends the improvement of roads in the regions where future hospital expansions will occur. For example, the FLNDP policy recommends significant investment in the roads near Butare in Years 1 and 2, ahead of the facility expansion in Butare that is planned to occur in Year 3. We observe that the FLNDP policies favor improving roads that are minor highways rather than unclassified and residential streets. In the Eastern province, road improvements made on the way to Kigali (small, central province) have a cascading impact on travel times, with patients located further from existing facilities having larger reductions in travel time, even when no facilities are built.

Figure 5 also illustrates the travel time required to access these facilities when road network improvements in the FLNDP policy are also made. Under both the FLNDP policy and the FLP policy, the southwest and northeast regions of the country experience the longest travel times in Year 1, before any facilities are expanded. However, we observe that under the FLNDP policy, these regions have large reductions in travel times that are directly attributable to the improvements to the road network at this time. For example, some populations see their travel times reduced by close to one hour due to the improvements to the road network alone in Year 1. In the rural northeast of the country, neither the FLNDP nor FLP recommend a facility expansion in the Eastern Province. However, some people in this province see their rainy season travel times reduce by 90 minutes, which is attributable to road network improvements alone.

**Fig. 7:**
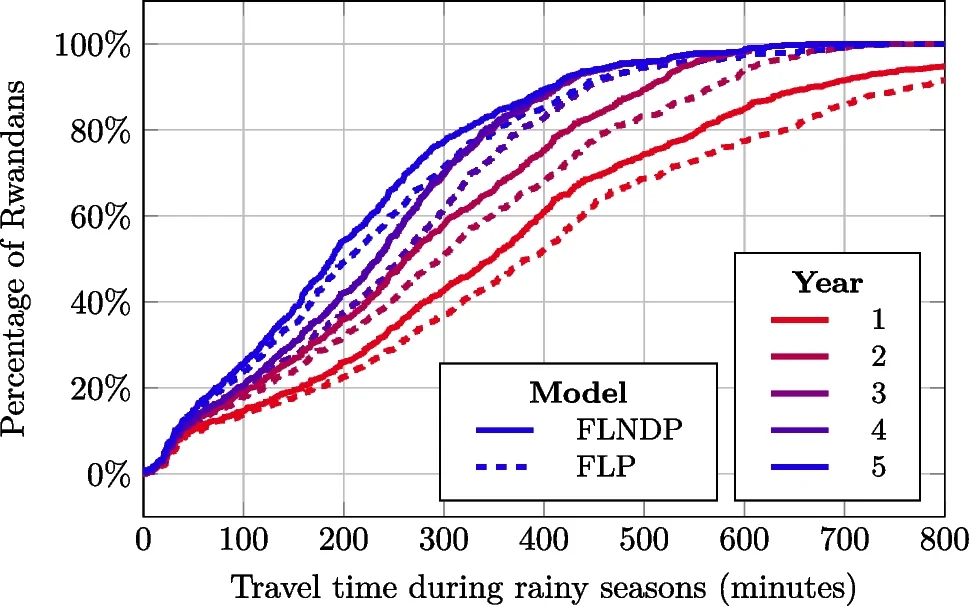
The distribution of travel time (in minutes) across the population over the planning horizon under both FLP and FLNDP policies (in representative test case 2)

Figure 6 illustrates where road investments are made and the final type of each arc at the end of the 5-year time horizon, in addition to the optimal expansion of facilities, in the reference scenario. We remark that all provinces would be expected to see investment in roads under the optimal expansion policy.

Figure 7 illustrates how the distribution of the population’s travel time during the rainy seasons changes over the planning horizon in our representative test case under both the FLP and FLNDP policies. We observe that the FLNDP policy improves access more quickly than the FLP policy. For instance, in Year 1, the FLP policy results in 20% of Rwandans traveling more than 10.8 hours to access care during the rainy seasons compared to 9.2 hours under the FLNDP policy. By Year 4, the FLNDP ensures that 80% of the population can access care in less than 5.7 hours during the rainy seasons. The FLP can achieve this level of access as well, but a year later, in Year 5.

**Fig. 8:**
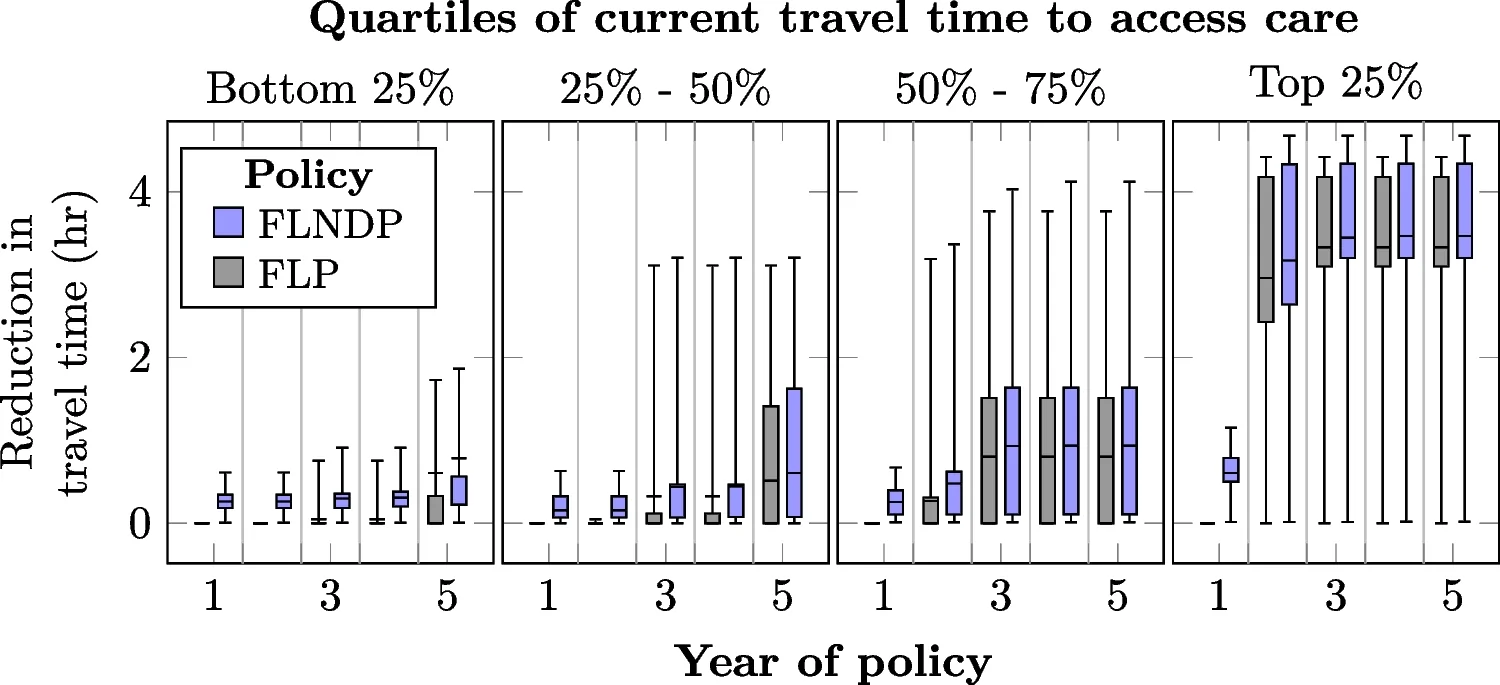
The distribution of reduction in travel time from baseline over the planning horizon under each policy in our representative test case (Table 6, test case 2), stratified by quartile of current travel times during the dry season. The bottom 25% quartile is comprised of the quarter of Rwandans who currently have the shortest travel times to access cancer care, while the top 25% quartile is comprised of the quarter of Rwandans who have the longest travel times to access care

Figure 8 illustrates the distribution of the reductions in travel time for each population center for each year of the planning horizon, relative to the current travel time for the respective population center. These distributions are shown for both the FLNDP policy and the FLP policy, stratified by quartile of current travel time. The bottom 25% quartile is comprised of the quarter of Rwandans who currently travel the least to access cancer care, while the top 25% quartile is comprised of the quarter of Rwandans who currently travel the farthest to access care. Figure 8 demonstrates that the FLNDP policy consistently provides higher reductions in travel time for each year and each quartile. In addition, we observe that Rwandans who currently have the worst access (the top 25% quartile) receive the largest reductions in travel time under both the FLP and FLNDP policies. Importantly, the FLNDP policy provides this group with larger travel time reductions, and these reductions are observed earlier in the planning horizon. For example, the FLNDP policy reduces the travel time for this quartile by 36 minutes on average in Year 1, compared to no reduction in travel time under the FLP policy in Year 1. In Years 2 through 5, the FLNDP policy consistently provides this quartile with larger travel time reductions than the FLP policy.

Across the majority of our test cases (Table 6), several structural trends generally hold. First, the planner should, whenever possible, add services to more hospitals. Secondly, the planner ought to prioritize high-value road improvements, both by surface and by location. In the above case study, graveling dirt roads offered a greater per-dollar increase in speed than paving gravel roads (Table 5). Thus, graveling high-traffic dirt roads would likely yield greater improvements than paving gravel roads. Further, these improvements can often proceed hospital construction. Lastly, in low-density regions, paving roads can offer meaningful reductions in travel time when population density does not support improving additional hospitals. While these trends may not hold in all instances, particularly those with parameters, demand, or existing road networks that are structurally different from Rwanda, we believe that they may provide useful managerial insights/rules of thumb without requiring the computational machinery presented within.

In summary, the FLNDP policy results in meaningful improvements in access compared to the FLP policy, especially for Rwandans who currently travel the farthest to access tertiary cancer care.

## Conclusions

In this article, we consider the problem of limited geographic access to cancer care in a low-income context and the extent to which improvements to transportation infrastructure could be used in concert with facility expansions to improve access.

### Overall summary

To address this challenge, we formulate a multi-period Facility Location Network Design Problem (FLNDP) with stochastic demand and travel times. During each period in our model, a central planner decides which facilities to expand and which roads to improve. Then, patients will select the shortest path to a facility offering cancer services given the road conditions due to weather. This formulation requires many fewer constraints than existing formulations in the literature. However, because there are a large number of possible paths, the resulting formulation requires a huge number of decision variables. To handle this, we propose a BP&C approach.

Within the BP&C algorithm, we use column generation to solve the linear programming relaxation at each node. In this step, we start by considering only a subset of path variables and iteratively generate new paths as needed by solving a pricing problem. We leverage the problem’s structure to efficiently solve the pricing problems by expressing them as shortest-path problems. Then, we tailor several modifications to accelerate our method, including heuristics and special ordered set branching rules that take advantage of the sequential decision periods. We also derive decomposition-based cuts that can be added at integer feasible solutions in the branch-and-bound tree. In a computational study, we compare our complete BP&C algorithm to a branch-and-price (B&P) algorithm that uses our proposed column generation approach, but does not include any cuts. We also compare BP&C to a VNS heuristic adapted from the literature. We find that the BP&C is able to solve more test instances than the B&P approach, suggesting that our proposed cuts provide meaningful improvements to the algorithm. We also find that our proposed model is most challenging to solve in instances with relatively tight monetary budgets. When compared to VNS, our BP&C algorithm is slower, but provides higher-quality solutions and reports an optimality gap.

In our case study, we explore geographic access to cancer care in Rwanda and compare two different approaches for expanding care: an FLP policy that only considers expansions to the healthcare infrastructure and an FLNDP policy that considers improvements to both the existing healthcare and the transportation infrastructure. The FLNDP policy consistently outperforms the FLP policy by further reducing travel times to access care due to its proposed road network improvements. In a representative test case, we observe that reductions in travel time that are directly attributable to road network improvements can be as high as one hour for some population centers. Moreover, we see that the patients with the largest reductions in travel time due to road network improvements are the patients who currently travel the farthest to access care.

### Avenues for future work

There are open opportunities for future work that builds on this work. Future technical work could further improve our BP&C approach. For instance, such work could strengthen our cuts via mixed-integer rounding, consider alternative branching schemes, or further improve the described cuts by considering the dual variables associated with existing cuts.

Future technical work could also consider applying the BP&C framework to different versions of the FLNDP. For example, the presented model could be extended to account for non-additive construction costs. Extensions of this model could consider capacitated facilities or adding equity considerations to expand the applicability of FLNDPs, but incorporating these features would make the problem more computationally challenging to solve. Yet another direction for future work would be to relax the assumption that patients always seek care at their nearest facility.

In reality, patients seek care depending on the quality of care offered, their trust in providers, travel distance, cost of care, and other factors. In Rwanda, work by Rubagumya et al. [39] demonstrated that financial toxicity presents a treatment barrier for many patients. If one facility offered services at a rate lower than other facilities, patients might be willing to travel further to receive services at lower cost. To our knowledge, there do not exist data capturing patient perceptions of these factors in Rwanda or similar contexts [51]. In the absence of these data, we made a simplifying assumption that patients seek care at their closest facility, which is commonly used in healthcare facility location-modeling [52, 53]. With a comprehensive registry of cancer patients, future work could incorporate higher-fidelity models of care-seeking behavior, such as discrete choice models, into the FLNDP [54–56].

A variant of the presented FLNDP with multiple types of facilities would enable modelers to better consider other diseases, such as HIV or diabetes, which require diagnosis and treatment at potentially separate facilities. Yet another opportunity for future work is the consideration of how increased geographic access to diagnosis could improve the rates of early diagnosis, which in turn may influence the demand for treatment.

Further, additional work could design policies that respond to observed uncertainty. Our model assumes a fixed construction policy chosen at the start of the planning horizon, before uncertainty is realized. Although this mirrors many long-term infrastructure plans, future research could adopt adaptive policies that adjust construction decisions as uncertainty is realized. Incorporating these features into a similar model would likely require a multi-stage stochastic program, which would be more computationally challenging to solve and may be more challenging to communicate with stakeholders.

Future modeling studies of Rwanda could consider more detailed weather estimates and include the ongoing costs of cancer care as these cost estimates become available. Further analysis of expected patient volumes at each expanded hospital might also be valuable, given that they are geographically distributed. Finally, because improved roads benefit many sectors beyond cancer care (as has been demonstrated by [57–59], and others), future work could allocate road improvement costs across both cancer-care and other stakeholders, including commercial, agricultural, and tourism sectors.

In summary, this article introduced a new method for expanding both healthcare and transportation infrastructure as a means to improve geographic access to cancer care. Our work demonstrates that improvements to the road network could meaningfully improve geographic access to cancer care, especially among cancer patients who are currently the most underserved. Our analysis provides valuable managerial insights to healthcare planners seeking to improve care access in low-income countries.

## Figures and Tables

**Fig. 9:**
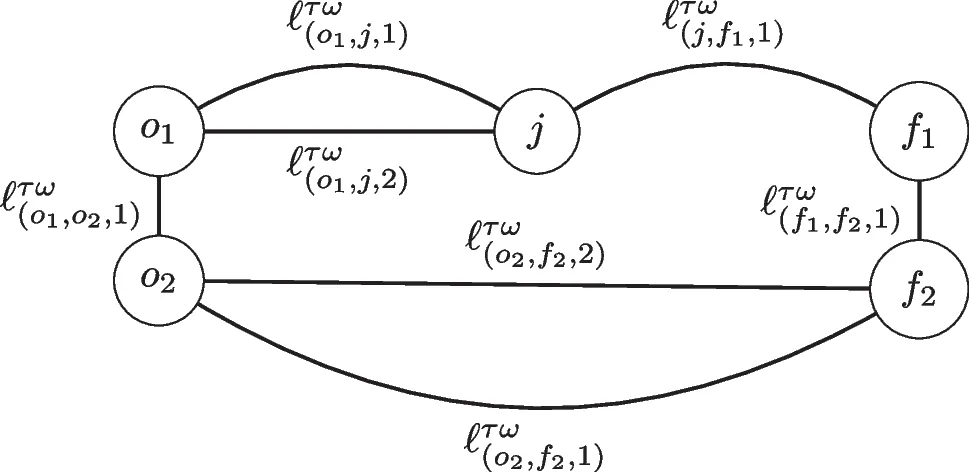
An example of a graph $\mathcal {G}$ with nodes $\mathcal {N}=\{o_1,o_2,j,f_1,f_2\}$, population centers $\mathcal {O}= \left\{ o_1,o_2\right\}$, candidate facilities $\mathcal {F}= \left\{ f_1,f_2\right\}$. The type-dependent arcs $\mathcal {A}$ are shown with their corresponding travel times

**Fig. 10:**
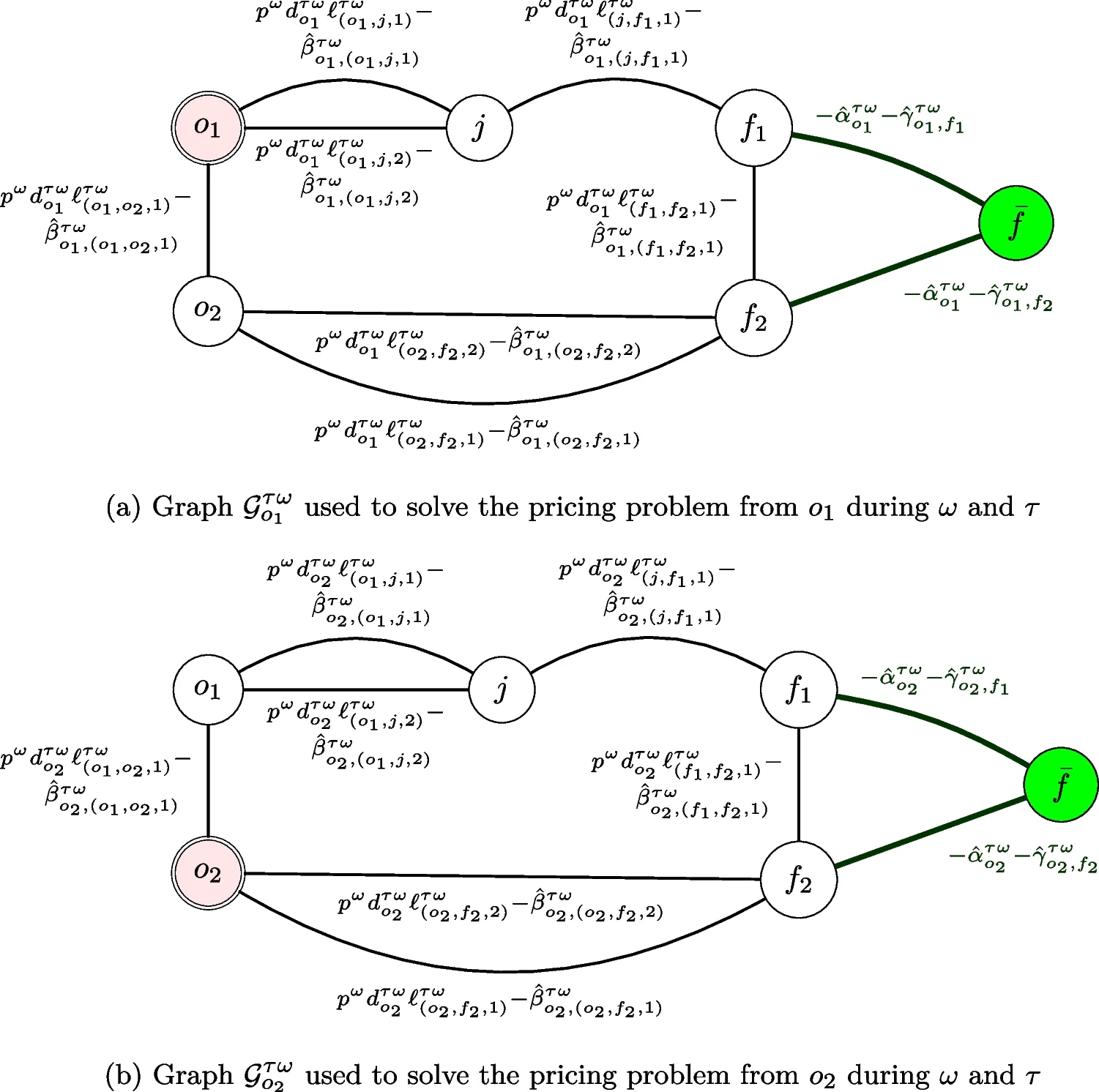
Examples of graphs to be used to solve the shortest-path problems corresponding to scenario ω and time period τ for two different population centers o, as described in §3.2. Figure 10a (resp. Figure 10b) illustrates the graph to be used to solve the shortest-path problem for $o= o_1$ (resp. $o= o_2$). In each graph, $\hat{\alpha }, \hat{\beta },$ and $\hat{\gamma }$ denote optimal dual values of the restricted master problem

**Fig. 11:**
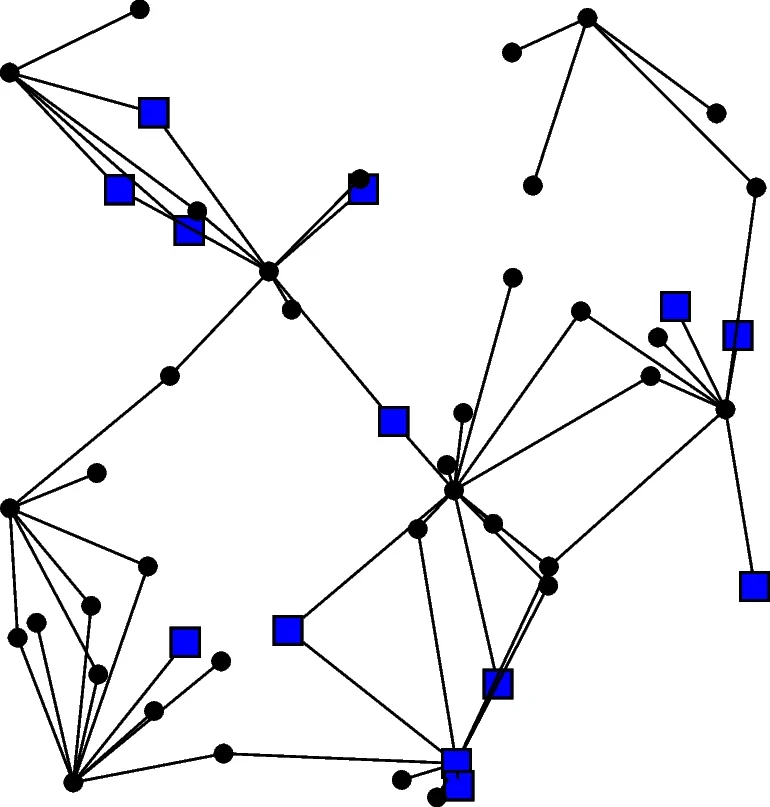
An example instance of a randomly constructed instance. Candidate facilities are shown as blue rectangles, with population centers shown as black circles. In this instance, $|\mathcal {N}| =50$ and $|\mathcal {F}|= 12$

**Algorithm 3:**
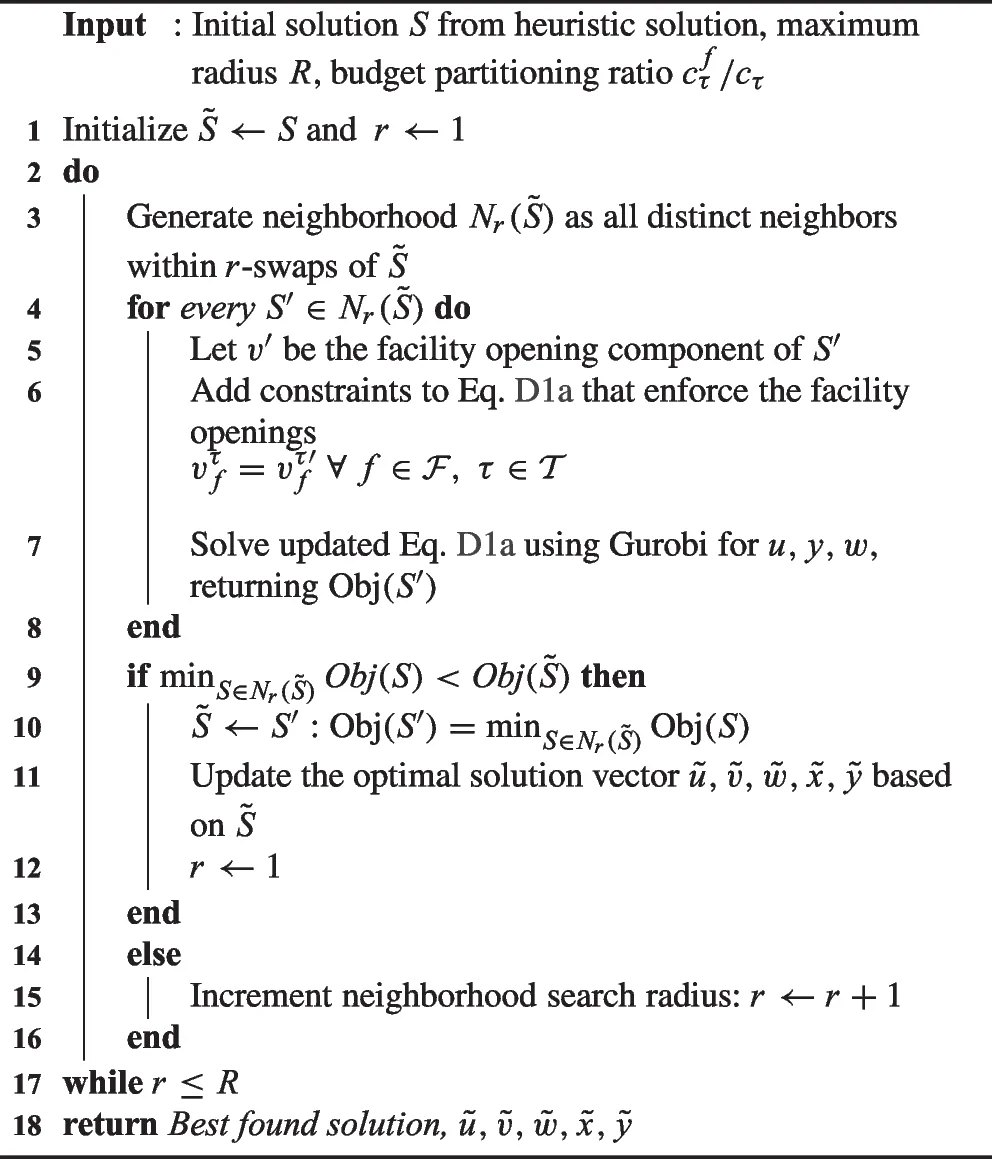
Hybrid variable neighbor search.

**Fig. 12:**
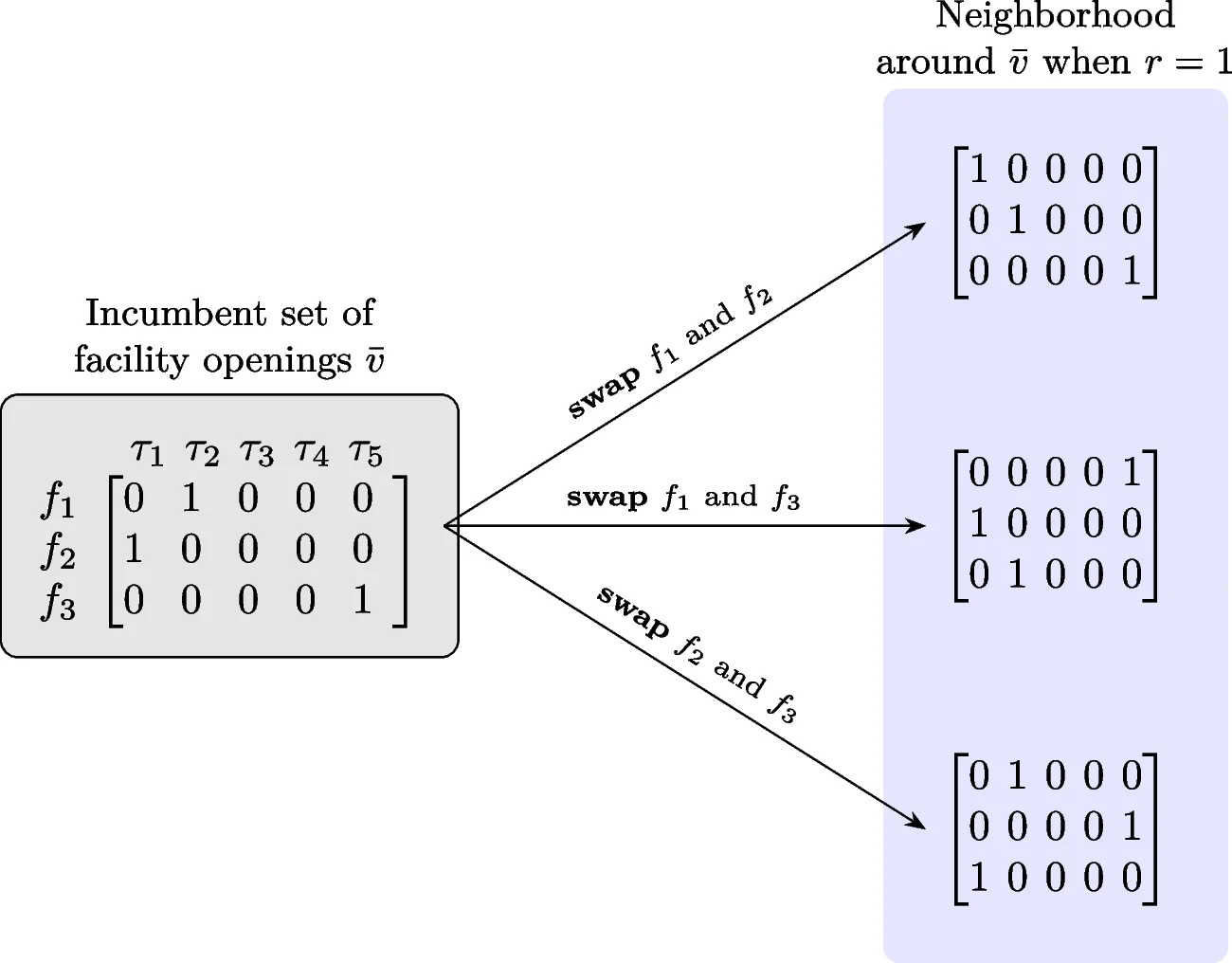
Swap procedure for generating neighborhoods of *similar* sets of open facilities around an incumbent set of locally optimal facility openings and timings given by $\bar{v}$. Here $\mathcal {F}= \left\{ f_1, f_2, f_3\right\}$ and T=5

## Data Availability

All data utilized in this study were obtained from publicly accessible open sources. The processed data supporting the findings of this study are available from the corresponding author upon reasonable request.

## Appendix A Author reflexivity statement

**Table:**

	Question	Response
	**Study Conceptualization**	
1.	How does this study address local research and policy priorities?	This modeling study creates a new algorithmic approach for facility location network design problems to improve geographic access in LMICs at nation scale. This algorithmic approach is demonstrated using a case study of Rwanda, addressing a national research interest of expanding access to cancer care and adds an advanced modeling dimension to the growing body of work on barriers to access to cancer care within Rwanda.
2.	How were local researchers involved in study design?	AS conceptualized the current modeling study when he was a visiting student at the University of Global Health Equity as a Watson Fellow. This fellowship supported AS’s time to collaborate with LMIC researcher Cristina Stefan. AS and LNS previously collaborated with LMIC author Cristina Stefan on a prior study for which AS collected publicly available data. The contributions of the current paper were focused on model formulation and algorithm development. AS made use of this previously collected data for the case study in this paper.
	**Research Management**	
3.	How has funding been used to support the local research team(s)?	No funding was provided.
	**Data acquisition and analysis**	
4.	How are research staff who conducted data collection acknowledged?	AS collected all data from public sources that are cited where appropriate and is the corresponding author. Vincent Cubaka provided cost estimates of hospital expansions and is thanked in the acknowledgments. Emmanuel Ngwakongnwi, Sage Ishimwe, and Cristina D. Stefan graciously hosted AS at the University of Global Health Equity and shared domain knowledge on cancer care in Rwanda and are thanked accordingly in the acknowledgments.
5.	How have members of the research partnership been provided with access to study data?	All data have been provided to the authors of both this article and the authors of the related prior study.
6.	How were data used to develop analytical skills within the partnership?	Data were not used to develop analytical skills within the partnership.
	**Data interpretation**	
7.	How have research partners collaborated in interpreting study data?	Cristina D. Stefan assisted in interpreting data for a prior study focused on access to cancer care in Rwanda. Dr. Stefan did not assist in interpreting any data for this study.

**Table:**

	Question	Response
	**Drafting and revising for intellectual content**	
8.	How were research partners supported to develop writing skills?	This study has not contributed to developing writing skills. All authors were extensively involved in manuscript writing.
9.	How will research products be shared to address local needs?	This article will be shared open-access. The expansion policies have been shared in advance of publication with the University of Global Health Equity.
	**Authorship**	
10.	How is the leadership, contribution, and ownership of this work by LMIC researchers recognized within the authorship?	The leadership, contribution, and ownership of this work did not include LMIC researchers, as the contributions of this work were focused on model formulation and algorithm development. These approaches are demonstrated on a case study of Rwanda that extends a prior study [8], which was conducted in partnership with LMIC researcher Dr. Stefan and for which she was recognized as last author.
11.	How have early career researchers across the partnership been included within the authorship team?	All 3 authors were early career researchers at the time the research was conducted.
12.	How has gender balance been addressed within the authorship?	2 authors identify as male and 1 as female.
	**Training**	
13.	How has the project contributed to training of LMIC researchers?	This project has not contributed to training of LMIC researchers.
	**Infrastructure**	
14.	How has the project contributed to improvements in local infrastructure?	This study has not contributed to improvements in local infrastructure.
	**Governance**	
15.	What safeguarding procedures were used to protect local study participants and researchers?	All analyses were performed on aggregate data describing physical infrastructure in Rwanda and are publicly available.

## Appendix B Additional details on branch-price-and-cut

### Appendix B.1 Pricing problem example

In this section, we provide an example on how to generate the graph $\mathcal {G}_{o}^{\tau \omega }$ used in the pricing problems corresponding to population center *o* in time period τ and scenario ω (as described in §3).

Figure 9 provides an example of a small graph $\mathcal {G}$ with nodes $\mathcal {N}= \{o_1,o_2,j,f_1,f_2\}$, population centers $\mathcal {O}= \left\{ o_1,o_2\right\}$, and candidate facilities $\mathcal {F}= \left\{ f_1,f_2\right\}$. The corresponding travel time along the arc between node *i* and node *k* of surface type s for this particular scenario ω and time period τ is indicated on the corresponding type-dependent arc a=(i,k,s) as $\ell _{a}^{\tau ,\omega }=\ell _{(i,k,s)}^{\tau ,\omega }$. Recall that $d_{o}^{\tau \omega }$ is the demand from population center o during time period τ and scenario ω, and $p^{ \omega }$ is the occurrence probability of scenario ω.

We now demonstrate how to construct the graph $\mathcal {G}_{o}^{\tau \omega }$ that is used to solve the pricing problem as a shortest-path problem corresponding to each population center o in scenario ω and time period τ. First, we create $\mathcal {G}_{o}^{\tau \omega }$ as a copy of $\mathcal {G}$ described above. We augment $\mathcal {G}_{o}^{\tau \omega }$ by adding a dummy sink node $\bar{f}$ and connecting it with each facility *f*. Then, we assign the length of each type-dependent arc in graph $\mathcal {G}_{o}^{\tau \omega }$ according to (5):

$$\begin{aligned} l_{a} = \left\{ \begin{array}{ll} {p^{ \omega }}d^{\tau \omega }_{ o}\ell ^{\tau \omega }_{a} - \hat{\beta }^{\tau \omega }_{oa} &  \text {if } ijs\in \mathcal {A}\\ - \hat{\alpha }^{\tau \omega }_{o} - \hat{\gamma }^{\tau \omega }_{of} &  \text {if } a = (f,\bar{f}) \text { for some } f\in \mathcal {F}, \end{array}\right. \end{aligned}$$

where $\hat{\alpha },\hat{\beta },$ and $\hat{\gamma }$ are obtained from solving the restricted master problem.

Figure 10 provides an example of such a graph for two different populations, $o_1$ and $o_2$, during scenario ω and time period τ. Figure 10a illustrates the graph to be used in the shortest-path problem for population $o_1$. Figure 10b illustrates how a similar process would be used to generate the graph corresponding to population $o_2$.

### Appendix B.2 Decomposition-based cuts

#### Appendix B.2.1 Non-dominated cuts

Here, we consider that some of the cuts of the form Eq. (8) may be dominated by previously generated cuts. Therefore, we provide an algorithm to generate a set of non-dominated cuts to be added to the master problem solved at each tree node. If the node which we are currently processing with column generation yields an integer solution, from which we can construct the cuts Eq. (8), we want to ensure that these cuts are not dominated by any pre-existing cuts to avoid slowing down the branch-price-and-cut algorithm. Thus, we compare the new cut, associated with $(\alpha ^{\tau \omega }_{o},(\beta ^{\tau \omega }_{oa})_{a \in \mathcal {A}},(\gamma ^{\tau \omega }_{of})_{f\in \mathcal {F}})$ with prior cuts associated with $(\alpha ^{\tau \omega ^\prime }_{o},(\beta ^{\tau \omega ^\prime }_{oa})_{a \in \mathcal {A}},(\gamma ^{\tau \omega ^\prime }_{of})_{f\in \mathcal {F}})$. The new cut is *dominated* by the old cut if

$$\begin{aligned} \min  &   \left\{ \alpha ^{\tau \omega ^{\prime }}_{o}- \alpha ^{\tau \omega }_{o}, \min _{a \in \mathcal {A}}\left\{ \beta ^{\tau \omega ^\prime }_{oa}\right. \right. \\  &   \left. \left. -\beta ^{\tau \omega }_{oa}\right\} , \min _{f\in \mathcal {F}}\left\{ \gamma ^{\tau \omega ^\prime }_{of}- \gamma ^{\tau \omega }_{of}\right\} \right\} \ge 0. \end{aligned}$$

If the new cut is not dominated by any of the existing cuts, then we add it to the cut set $\mathcal {C}$.

#### Appendix B.2.2 Generating cuts from heuristic integer solutions

We now describe our process for generating valid inequalities (8) when we only have access to integer-feasible first-stage solutions ($\hat{u},\hat{v},\hat{x},\hat{y}$). This would permit us to generate cuts from the heuristic integer solution in §3.4.1 without having access to dual variables at optimality of the master problem. Recall that given binary first-stage variables, all second-stage decisions result from solving shortest-path problems. Thus, this problem bears structural similarity to that of Magnanti et al. [28] and Forbes [23], which we now extend to consider our specific FLNDP in which there are multiple surface types, $\mathcal {S}$. The dual variables have the following natural interpretations for a fixed τ and fixed ω. $\gamma ^{\tau \omega }_{of}$ is the change in travel time during ω for o if f were to have been constructed at the start of τ. Similarly, $\beta _{o(i,k,s)}^{\tau \omega }$ denotes the change in travel time during ω for o if (*i*, *k*, *s*) were to have been constructed at the start of τ. Lastly, $\alpha _{o}^{\tau \omega }$ is the travel time currently incurred by o in τ under ω.

Since $\hat{u},\hat{v},\hat{x},\hat{y}$ are integer, we create a subgraph $\overline{\mathcal {G}}^{\tau \omega }:= (\mathcal {N}, \overline{\mathcal {A}}^{\tau \omega })$ for each $\tau \in \mathcal {T}$ and $\omega \in \Omega$ by including only the arcs that are available at the start of period τ, namely, $\overline{\mathcal {A}}^{\tau \omega } := \left\{ (i,k,s) \in \mathcal {A}\text { s.t. }\hat{y}^{\tau }_{a} = 1\right\}$. Then, for $o\in \mathcal {O}$, we compute the travel time to all other nodes in $\overline{\mathcal {G}}^{\tau \omega }$ using $\ell ^{\tau \omega }$ as arc lengths. Following the convention of Pearce and Forbes [23], let $D_{on}$ denote the shortest travel time to any node $n \in \mathcal {N}$ from o. Then $\alpha ^{\tau \omega }_{o} = \min \left\{ D_{of}, \ \forall \ f\in \mathcal {F}\ \text { s.t. }\ \hat{x}^{\tau }_{f} = 1\right\}$. Let $\Delta _{n} := \min \left\{ 0,D_{on} - \alpha ^{\tau \omega }_{o}\right\}$. Note that $\Delta _n$ is equal to 0 when *n* is located further than the closest existing facility and is negative when *n* is closer than such a facility. Thus we can define the remaining dual variables based on their intuited definitions:

$$\begin{aligned} \gamma ^{\tau \omega }_{of}&= \Delta _{f}, \ \forall \ f\in \mathcal {F}\end{aligned}$$

$$\begin{aligned} \beta ^{\tau \omega }_{o(a)}&\!=\! \min \left\{ 0, \underbrace{\min \left\{ \Delta _i, \Delta _k\right\} }_{\text {close endpoint}} - \underbrace{\max \left\{ \Delta _i,\Delta _k\right\} }_{\text {far endpoint}} + \ell ^{\tau \omega }_{(a)}\right\} \end{aligned}$$

Briefly, $\beta ^{\tau \omega }_{o(a)}$ is obtained by considering the difference in Δ between the endpoint of (*a*) located closest to o and the far endpoint of (*a*). This difference expresses the reduction in travel time if no time was required to traverse (*a*). To this difference, we add the travel time $\ell ^{\tau \omega }_{ijs}$ required to traverse. Without altering $\overline{\mathcal {G}}^{\tau \omega }$, we can repeat the process for each $o\in \mathcal {O}$. To solve for all dual variables, we must construct a new $\overline{\mathcal {G}}^{\tau \omega }$ for each $\tau \in \mathcal {T}$ and $\omega \in \Omega$. We use these dual variables to construct cuts of the form Eq. (8).

## Appendix C Additional details of computational experiments

In this appendix, we provide more details about the randomly generated instances we used to test our solution algorithms. We generate the nodes $\mathcal {N}$ and the candidate facilities as described in §4. We also define the time periods $\mathcal {T}$ and scenarios Ω. Now, we provide more details on how each instance is generated given $\mathcal {N}, \mathcal {F}, \mathcal {T},$ and Ω.

### Appendix C.1 Road network construction

First, we describe how we constructed the road networks available at the beginning of the planning horizon. Our construction process mimics the reality that road networks are often sparse, with the degree of the most connected node rarely exceeding 6. Further, our process models that the closer two nodes are in an observed road network, the more likely they are to be connected. Our first step is to calculate the Euclidean distance between each pair of nodes in the network. Then, we select a random node in the network, $$i\in \mathcal {N}$$. For this node *i*, we rank the other nodes in the network according to their distance from *i*. Therefore, for node *i*, we can label the other nodes such that $$i_1$$ is the closest node to *i* and $$i_{|\mathcal {N}|-1}$$ is the farthest node from *i*. Finally, we randomly generate an arc between *i* and $$i_n$$ with probability $$\frac{1}{n}$$ for all $$n \in \{1,\dots ,|\mathcal {N}|-1\}$$. We then ignore node *i* when considering the generation of future arcs and select a new node $i^{\prime }$. We repeat this process where we now rank the $$|\mathcal {N}|-2$$ nodes in $$\mathcal {N}\setminus \{i,i^\prime \}$$ and generate arcs between the remaining nodes with the probability described above (i.e., the arc between $i^{\prime }$ and $$i'_n$$ is generated with probability $$\frac{1}{n})$$. This process guarantees we will construct a connected graph. In addition, this process generates graphs in which nodes that are closer to each other are more likely to have an arc between them, which is characteristic of road networks. Figure 11 illustrates an example of a randomly generated graph using this process.

Next, we define the type of surface for each arc (*i*, *j*). We consider $|\mathcal {S}|=5$ different surface types. The probability that an arc (*i*, *j*) will take on surface type s is given by a truncated, shifted geometric distribution:

$$\begin{aligned} p(\text {surface}(i,j) \!=\! s) \!=\! \left\{ \!\begin{array}{ll} 1\!-\!\frac{1}{|\mathcal {S}|}\sum _{s'=2}^{|\mathcal {S}|}\!\left( 1\!-\!\frac{1}{|\mathcal {S}|}\right) ^{s'-1}, &  \text { if }s = 1,\\ \frac{1}{|\mathcal {S}|}\left( 1\!-\!\frac{1}{|\mathcal {S}|}\right) ^{s-1}, &  \text {if } s \!\in \! \{2,\ldots ,|\mathcal {S}|\}. \end{array}\right. \end{aligned}$$

This distribution is selected to model reality. In many LICs, most roads are of poor quality, with dirt roads being more common than gravel, gravel being more common than chip-sealed, and chip-sealed roads being more common than paved highways. The truncated geometric distribution generates synthetic networks with this property. At this point, we have now generated the nodes $\mathcal {N}$, candidate facilities $\mathcal {F}$, time periods $\mathcal {T}$, scenarios Ω, and the existing road network. We now describe how we generate the remainder of the parameters for each instance.

### Appendix C.2 Monetary and human capital cost parameters

We assume a uniform monetary cost of constructing arcs $c_{ijs}^{\tau \omega }$ and adding services ${c_{f}}^{\tau }$ across time periods $\tau \in \mathcal {T}$. The cost associated with constructing arc (*i*, *j*, *s*) is a function of $\Vert i-j\Vert _2$, the Euclidean distance from *i* to *j*, the surface condition *s*, as well as the already existing surface condition $s^{\prime }$ connecting *i* and *j*.

We set that

$$\begin{aligned} c_{ijs}^{\tau } = \left\{ \begin{array}{ll} \Vert i-j\Vert _2 \cdot \frac{1}{10}s, &  s > s^{\prime }, \\ 0, &  s \le s^{\prime }. \end{array}\right. \end{aligned}$$

where ||i-j||2 is the ℓ2 norm between the arcs endpoints.

This relationship was based upon Sibomana and Ngendahimana [45], who note both distance and surface dependencies in arc construction costs. Other factors, such as the grade and drainage of the arc may impact the construction cost, but these were not incorporated into our simulated instances.

The cost associated with a single facility ${c_{f}}^{1}$ was distributed according to a truncated normal distribution centered at 75 with a standard deviation of σ=5 truncated to the interval (50, 100). These costs are assumed to be consistent across the horizon, i.e., $c_{f}^{\tau }={c_{f}}^{1},\;\forall \tau \in \mathcal {T}$. We selected this distribution to mimic the reality that construction costs are generally similar, as building a similar sized facility is likely to cost a similar amount, but there may be individual site variation in real cost. The distribution was truncated to avoid erroneously expensive facilities. Staffing costs for each hospital were also assumed to be consistent across time periods, with ${h_{f}}^{1} \sim \text {Unif}(2,6)$ and ${h_{f}}^{\tau }={h_{f}}^{1},\;\forall \tau \in \mathcal {T}$. This distribution was selected based on [8] who estimated the number of oncologic teams required for each hospital expansion.

#### Appendix C.2.1 Demand parameters

We consider that all nodes are population centers. We assume that the demand for a fixed population center $o\in \mathcal {O}$ is independent of the scenarios, but varies across time periods $\tau \in \mathcal {T}$. Demand is drawn from a temporally correlated distribution that grows over each time period, to reflect a growing population and population-dependent demand. Demand for each population center $o\in \mathcal {O}$ during the first time period is generated according to $d^{1\omega }_{o} \sim \text {Unif}(50,100)$. Demand in all other time periods, i.e., $\tau \ne 1$, is generated for each population center $o\in \mathcal {O}$ such that $d^{\tau \omega }_{o} \sim d^{(\tau -1) \omega }_{o} + \text {Unif}(25,50)$.

#### Appendix C.2.2 Travel time parameters

We specify the travel time $\ell _{ijs}^{\tau \omega }$ for type-dependent arc $(i,j,s)\in \mathcal {A}$, in each of the two cases $\omega _\tau \in \{\text {dry},\text {rainy}\}$ to be the same in all time periods $\tau \in \mathcal {T}$. We specify $\ell _{ijs}^{\tau \omega }$ as follows:

$$\begin{aligned} \ell _{ijs}^{\tau \omega } = \frac{\Vert i-j\Vert _2}{s+1}\cdot \left( 1 + \frac{1}{2} {1\!\!1\{\omega _\tau = \text { rainy}\}}\right) \end{aligned}$$

## Appendix D Heuristic comparison: variable neighbor search

To compare the performance of our BP&C algorithm with other existing literature approaches, we extended the edge-based formulation from Pearce and Forbes [23] to the stochastic problem with multiple edge types. A key difference is that our problem relies upon a single monetary budget C, for both arc improvements and facility improvements, whereas prior literature uses two monetary budgets, one for arc improvements and one for facility improvements. Let $C_{\mathcal {A}}^{\tau }$ and $C_{\mathcal {F}}^{\tau }$ be the budgets associated with arc improvements and facility improvements, respectively.

We use the notation defined in Tables 1 and 2. However we also introduce a new decision variable, $w_{oa}^{\tau \omega }$, which indicates the fraction of demand originating from $o\in \mathcal {O}$ that traverses arc $a \in \mathcal {A}$ in $\omega \in \Omega$ during time period $\tau \in \mathcal {T}$. Additionally, we now assume that arcs are directed and define the inbound arcs, $\delta ^+_n = \left\{ (i,n,s) \in \mathcal {A}\right\}$, and outbound arcs, $\delta ^-_n = \left\{ (n,j,s) \in \mathcal {A}\right\}$, for each node $n \in \mathcal {N}$. We formulate the problem as:

D1a $$\begin{aligned}&\underset{u,v,w,x,y}{\text {minimize}}{ \sum _{\omega \in \Omega }p^{ \omega } \sum _{\tau \in \mathcal {T}}\sum _{o\in \mathcal {O}}d_{o}^{\tau \omega } \sum _{a \in \mathcal {A}} \ell _{a}^{\tau \omega } w_{oa}^{\tau \omega }} {} \end{aligned}$$

D1b $$\begin{aligned}&\text {subject to}~(\text {1c})-(\text {1h}) \nonumber \\&{\sum _{\tau =1}^{\tau ^{\prime }}\left( \sum _{a \in \mathcal {A}} c^{\tau }_{a} v^{\tau }_{a}\right) }{\le \sum _{\tau =1}^{\tau ^{\prime }}C^{\tau }_{\mathcal {A}} \quad \forall \ \tau ^{\prime } \in \mathcal {T},} \end{aligned}$$

D1c $$\begin{aligned}&{\sum _{\tau =1}^{\tau ^{\prime }}\left( \sum _{f\in \mathcal {F}} c^{\tau }_{f}u^{\tau }_{f}\right) }{\le \sum _{\tau =1}^{\tau ^{\prime }}C^{\tau }_{\mathcal {F}} \quad \forall \ \tau ^{\prime } \in \mathcal {T},} \end{aligned}$$

D1d $$\begin{aligned}&{C^{\tau }_{\mathcal {A}} + C^{\tau }_{\mathcal {F}}}{\le C^{\tau } \quad \forall \ \tau \in \mathcal {T}, } \end{aligned}$$

D1e $$\begin{aligned}&{\sum _{a \in \delta ^{-}_{o}}w_{oa}^{\tau \omega }}{\ge 1 \ \forall \ o\in \mathcal {O}\setminus \mathcal {F}, \omega \in \Omega , \tau \in \mathcal {T} ,} \end{aligned}$$

D1f $$\begin{aligned}&{\sum _{a \in \delta ^-_{f}}w_{fa}^{\tau \omega } + x_{f}^{\tau } }{\ge 1 \ \forall \ f\in f\cap \mathcal {O}, \omega \in \Omega , \tau \in \mathcal {T}, } \end{aligned}$$

D1g $$\begin{aligned}&{\sum _{a \in \delta ^{-}_{n}}w_{oa}^{\tau \omega } \!-\! \sum _{a \in \delta ^{+}_{n}} w_{oa}^{\tau \omega }}{ \!=\! 0 \ \forall \ n \!\in \! \mathcal {N}\setminus \left\{ \mathcal {F}\cup \left\{ o\right\} \right\} , o\!\in \! \mathcal {O}, \omega \!\in \! \Omega , \tau \!\in \! \mathcal {T}, } \end{aligned}$$

D1h $$\begin{aligned}&{\sum _{a \in \delta ^{+}_{f}}w_{oa}^{\tau \omega } - \sum _{a \in \delta ^{-}_{f}} w_{oa}^{\tau \omega }}{ \le x_{f}^{\tau } \ \forall \ f\in \mathcal {F}\setminus \left\{ o\right\} , o\in \mathcal {O}, \omega \in \Omega , \tau \in \mathcal {T}, } \end{aligned}$$

D1i $$\begin{aligned}&{w_{oa}^{\tau \omega }}{\le y_{a}^{\tau \omega }\ \forall \ o\in \mathcal {O}, a \in \mathcal {A}, \omega \in \Omega , \tau \in \mathcal {T}, } \end{aligned}$$

D1j $$\begin{aligned}&{0 \le w_{oa}^{\tau \omega }}{\le 1 \ \forall \ o\in \mathcal {O}, a \in \mathcal {A}, \omega \in \Omega , \tau \in \mathcal {T}. } \end{aligned}$$

Equation (D1b) enforces that the budget spent on arc improvements cannot exceed that allocated for such improvements and Eq. (D1c) enforces the same condition for facilities. Equation (D1d) states that, at each time period, the budget allocated to arc improvements and the budget allocated for facilities cannot exceed the total monetary budget. Equation (D1e) states that all demand for services must exit its origin along an outbound edge, with Eq. (D1f) considering that improved facilities can meet their own demand. Equations (D1g) and (D1h) enforce that the flow from each origin into another nodes must be equal to the flow out of that node, unless the node is an improved facility. Constraint Eq. (D1i) limits flow to improved arcs. Equation (D1j) states that the flow from a particular origin along all arcs must be between zero and one.

### Appendix D.1 Hybrid variable neighbor search to the arc-based stochastic formulation

We now describe the VNS approach to which we compare our solution methodology in §4. Both Cocking [37] and Ghaderi [22] make use of VNS to solve related FLNDPs. We adapt the approach described by [22], with modifications to the algorithm described in [22]’s Fig. 2. As input, we take both an FLNDP instance and an input parameter indicating the proportioning of the budget, ${C_{f}^{\tau }}/{C^{\tau }}$. We construct a vector representation of a policy, $\bar{S}$, by considering only the *u* and *v* binary variables. We then generate *v* vectors that are similar in their facility improvements to the incumbent optimal solution using a *swap* mechanism. We perform a local search by using Gurobi to solve for the optimal network improvements at each *v* vector, constructing a new pool of candidate policies.

Like our heuristic proposed in §3.4.1, VNS begins by constructing a partial initial solution considering only facility improvements. This generates an initial vector $\bar{v}$. We complete the initial solution, $\bar{S}$, by using Gurobi to solve Eq. ((D1a)) under the additional constraint $v_{f}^{\tau } = \bar{v}_{f}^{\tau }$ for all $f\in \mathcal {F}, \ \tau \in \mathcal {T}$. To produce a neighborhood of radius *r* around $\bar{S}$, we generate all possible $v^{\prime }$ resulting from *r*-swaps from $\bar{v}$. A swap interchanges two facilities opening times in $\bar{v}$ such that $v_{f_1}^{\tau \prime } = \bar{v}_{f_2}^{\tau }$ and $v_{f_2}^{\tau \prime } = \bar{v}_{f_1}^{\tau }$ for each $\tau \in \mathcal {T}$. This can alternatively be thought of interchanging rows in a matrix representation, as shown in Fig. 12. At radius *r*, the Hamming distance between $\bar{v}$ and any possible $v^{\prime }$ is at most 2*r*.

For each $v^{\prime }$, the corresponding solution, $S^{\prime }$, is again completed by solving Eq. (D1a) with facility improvements fixed to $v^{\prime }$. To avoid re-solving $v^{\prime }$, which may be re-generated at a different *r* by reversing the row interchanges, we maintain a list of *v* that have previously been solved. This is formalized in Algorithm 3. The algorithm terminates either when a maximal search radius *R* is reached or a time limit is reached.
